# *Pseudomonas fluorescens* MFE01 uses 1-undecene as aerial communication molecule

**DOI:** 10.3389/fmicb.2023.1264801

**Published:** 2023-10-16

**Authors:** Charly A. Dupont, Yvann Bourigault, Théo Osmond, Maëva Nier, Corinne Barbey, Xavier Latour, Yoan Konto-Ghiorghi, Julien Verdon, Annabelle Merieau

**Affiliations:** ^1^Laboratoire de Communication Bactérienne et Stratégies Anti-infectieuses (CBSA UR), Univ Rouen Normandie, Université Caen Normandie, Normandie Univ, Rouen, France; ^2^Structure Fédérative de Recherche Normandie Végétale and Entente Franco-Québécoise NOR-SEVE, NORVEGE, Rouen, France; ^3^Laboratoire Ecologie and Biologie des Interactions, Université de Poitiers, Poitiers, France

**Keywords:** bacterial communication, *Pseudomonas fluorescens* MFE01, volatile compounds, biofilm, type six secretion system

## Abstract

Bacterial communication is a fundamental process used to synchronize gene expression and collective behavior among the bacterial population. The most studied bacterial communication system is quorum sensing, a cell density system, in which the concentration of inductors increases to a threshold level allowing detection by specific receptors. As a result, bacteria can change their behavior in a coordinated way. While in *Pseudomonas* quorum sensing based on the synthesis of *N*-acyl homoserine lactone molecules is well studied, volatile organic compounds, although considered to be communication signals in the rhizosphere, are understudied. The *Pseudomonas fluorescens* MFE01 strain has a very active type six secretion system that can kill some competitive bacteria. Furthermore, MFE01 emits numerous volatile organic compounds, including 1-undecene, which contributes to the aerial inhibition of *Legionella pneumophila* growth. Finally, MFE01 appears to be deprived of *N*-acyl homoserine lactone synthase. The main objective of this study was to explore the role of 1-undecene in the communication of MFE01. We constructed a mutant affected in *undA* gene encoding the enzyme responsible for 1-undecene synthesis to provide further insight into the role of 1-undecene in MFE01. First, we studied the impacts of this mutation both on volatile organic compounds emission, using headspace solid-phase microextraction combined with gas chromatography-mass spectrometry and on *L. pneumophila* long-range inhibition. Then, we analyzed influence of 1-undecene on MFE01 coordinated phenotypes, including type six secretion system activity and biofilm formation. Next, to test the ability of MFE01 to synthesize *N*-acyl homoserine lactones in our conditions, we investigated *in silico* the presence of corresponding genes across the MFE01 genome and we exposed its biofilms to an *N-*acyl homoserine lactone-degrading enzyme. Finally, we examined the effects of 1-undecene emission on MFE01 biofilm maturation and aerial communication using an original experimental set-up. This study demonstrated that the Δ*undA* mutant is impaired in biofilm maturation. An exposure of the Δ*undA* mutant to the volatile compounds emitted by MFE01 during the biofilm development restored the biofilm maturation process. These findings indicate that *P. fluorescens* MFE01 uses 1-undecene emission for aerial communication, reporting for the first time this volatile organic compound as bacterial intraspecific communication signal.

## 1. Introduction

Bacteria exchange chemical signals to communicate with each other (Miller and Bassler, 2001). These signals are used to coordinate gene expression and collective behaviors, including those involved in virulence, at a community level. Consequently, understanding bacterial communication is of the greatest interest for the development of new strategies to control bacterial populations and preventing infections (Waters and Bassler, 2005).

The most studied bacterial communication system is quorum sensing (QS), defined as a population-density-responsive gene regulation system allowing bacteria to sense population density and to act only when a quorum of cells is reached (Waters and Bassler, 2005). The chemical signals used in QS (i.e., autoinducers) are small molecules released and accumulated into the environment by bacteria. The concentration of autoinducers increases with bacterial growth until a threshold level is reached. At this point, these signals molecules are detected by specific receptors and their binding activates positive feedback and change in gene expression, allowing bacteria to adjust their behavior accordingly (Fuqua et al., 1994). In some *Pseudomonas*, QS is mainly based on the synthesis of *N*-acyl homoserine lactone molecules (AHLs) and their perception by a dedicated sensor (Papenfort and Bassler, 2016). In *Pseudomonas aeruginosa* a QS system based on alkyl-quinolone is also present (Papenfort and Bassler, 2016).

QS is a key factor for biofilm development, notably in *P. aeruginosa* (De Kievit, 2009). Biofilms are complex communities of microorganisms embedded in a matrix of extracellular polymeric substances (EPS) (Flemming et al., 2016). These complex structures are commonly found in natural and man-made environments and can have both beneficial and harmful effects, depending on the context (Hall-Stoodley et al., 2004). In addition to biofilm formation, QS regulates other behaviors such as motility or type six secretion systems (T6SSs) (Papenfort and Bassler, 2016; Gallique et al., 2017a).

T6SSs are nanomachines that use a contractile mechanism to propel an effector-loaded needle into target cells (Brackmann et al., 2017; Cherrak et al., 2019; Coulthurst, 2019; Wang et al., 2019). In most *Pseudomonas*, a functional T6SS requires at least 14 core proteins, which assemble onto a membrane complex and a tail structure (Brackmann et al., 2017; Cherrak et al., 2019; Coulthurst, 2019). The tail structure is made of a needle wrapped by a contractile sheath (Basler et al., 2012). The needle comprises the inner tube constituted of Hcp protein hexamers and by a spike complex made of a trimer of VgrG sharpened by PAAR protein that serves as prey cell penetrating device (Leiman et al., 2009; Shneider et al., 2013). The sheath is made of the TssB and TssC subunits and its contraction leads to the expulsion of the needle carrying T6SS effectors (Kapitein and Mogk, 2013). T6SSs are capable to inject a broad repertoire of toxins as anti-bacterial specific effectors, anti-eukaryotic specific effectors or trans-kingdom effectors (Hernandez et al., 2020; Monjarás Feria and Valvano, 2020; Jurėnas and Journet, 2021). In environmental *Pseudomonas,* T6SS plays an essential role in interbacterial competition, allowing bacteria to acquire extracellular elements such as DNA, metallic ions, and participates to bacteria communication (Ho et al., 2014; Hachani et al., 2016; Gallique et al., 2017a).

Volatile compounds (VCs), including volatile organic compounds (VOCs) are also considered to be communication signals within the rhizosphere (Ninkovic et al., 2021). VOCs are byproducts of the primary and secondary metabolism. These molecules are small (molecular weight <500 Daltons), with a high vapor pressure, low boiling point, and a lipophilic part. These physicochemical properties enable these molecules to be in the gaseous state under environmental conditions (Schmidt et al., 2015). Numerous studies have reported interaction between bacteria with plants, fungi, or other bacteria via VOCs emission (Kim et al., 2013; Létoffé et al., 2014; Schmidt et al., 2015; Raza et al., 2016; Netzker et al., 2020; Weisskopf et al., 2021; Vlot and Rosenkranz, 2022).

The *Pseudomonas fluorescens* MFE01 strain is able to protect potato-tuber from soft-rot against the phytopathogen *Pectobacterium atrosepticum* via its T6SS (Smadja et al., 2004; Decoin et al., 2014). Like in other *P. fluorescens* strains, no AHL-based QS system has been detected in MFE01 using biosensor-based assays (Wei and Zhang, 2006; Martins et al., 2014; Gallique et al., 2017b). Nevertheless, MFE01 strain coordinates its population during biofilm formation and swimming when its T6SS is functional (Decoin et al., 2015; Gallique et al., 2017b; Bouteiller et al., 2020). Previous works showed that MFE01, like other *Pseudomonas*, emits a broad range of VOCs (Corre et al., 2021). Among the VOCs emitted on lysogenic broth (LB) medium, MFE01 generates a high amount of 1-undecene which exerts long-range growth inhibition against *Legionella pneumophila*, the etiological agent of legionellosis (Bigot et al., 2013; Corre et al., 2021). A transposition mutant from this previous study suggested a link between T6SS activity and 1-undecene emission. Unfortunately, polar effects caused by transposon insertion did not allow to confirm these observations (Corre et al., 2021).

In this study, we constructed a mutant affected in *undA* gene encoding the enzyme responsible for 1-undecene synthesis to provide further insight onto the physiological role of 1-undecene in MFE01. First, we studied impact of this mutation on MFE01 VOCs emission, using headspace solid-phase microextraction combined with gas chromatography-mass spectrometry (HS-SPME/GC-MS). Then, the *undA* mutation effect was investigated on *L. pneumophila* long-range growth inhibition. We also analyzed influence of 1-undecene on the coordinated phenotypes of MFE01, including biofilm formation and T6SS antibacterial activity. Next, to confirm the inability of MFE01 to produce AHLs, we investigated *in silico* the presence of corresponding genes across its genome and we exposed its biofilms to an AHLs-degrading enzyme. Finally, we characterized the effect of 1-undecene emission on the maturation of MFE01 biofilms.

## 2. Materials and methods

### 2.1. Bacterial strains, plasmids, culture conditions and chemicals

All strains and plasmids used in this study are listed in Supplementary Table S1. *P. fluorescens*, *P. aeruginosa*, *Escherichia coli* and *P. atrosepticum* strains were grown in lysogenic broth (LB) medium and *L. pneumophila* lens strain in buffered yeast extract medium or on buffered charcoal yeast extract (BCYE) agar plates (Corre et al., 2019). *P. fluorescens* strains were grown at 28°C, *P. atrosepticum* strain at 25°C, *P. aeruginosa*, *E. coli* and *L. pneumophila* lens strains at 37°C. All liquid cultures were grown under constant rotary shaker at 180 rpm. Media were supplemented with antibiotics, as appropriate: 15 μg/mL tetracycline; 20 μg/mL or 50 μg/mL gentamycin for *E. coli* or *P. fluorescens*, respectively. Cultures carrying pJN105 plasmid were supplemented with 0.2% L-arabinose for gene expression. All chemicals were purchased from Sigma-Aldrich (St. Louis, MO, United States) unless otherwise stated.

### 2.2. General molecular biology procedures

PCR reactions were performed under standard conditions using Phusion^®^ high-fidelity DNA polymerase (NEB) in high fidelity buffer. The temperature of primer hybridization was calculated using NEB Tm calculator.^1^ Primers listed in Supplementary Table S2 were purchased from Eurogentec, Belgium. Restriction enzymes and T4 DNA ligase were used according to manufacturer’s instructions. Genomic DNA extraction and purification were performed using the “Genejet Genomic DNA purification Kit” (Thermo Fisher). PCR products were purified using the “PCR purification Kit” (Qiagen) or the “Gel purification Kit” (Qiagen) from agarose gels. All kits were used accordingly to manufacturers’ instructions.

### 2.3. Construction of the MFE01 Δ*undA* mutant strain

The markerless *undA* in-frame deletion mutant (Δ*undA*) contains a deletion of 762 base pairs (bp) in the *undA* gene. To obtain this deletion, the upstream and downstream regions of *undA* were amplified by PCR with the two primer pairs M1-*undA*/M2QC-*undA* (977 bp) and M3QC-*undA*/M4-*undA* (1,146 bp) respectively. Amplicons were used in overlap-PCR and re-amplified using M1-*undA*/M4*-undA* primers. The deleted *undA* gene construction was introduced into pAKE604 suicide vector (El-Sayed et al., 2001) digested by SmaI via blunt-ended ligation using T4 DNA ligase (NEB) and transformed in *E. coli* top 10 strain (Thermo Fisher Scientific). This construction was verified by Sanger-sequencing (Genewiz, Germany) and introduced into *E. coli* S17.1 strain (Simon et al., 1983). This plasmid was transferred in MFE01 by biparental mating as described by Bouteiller et al. (2020), and double recombination event was selected. The *in frame undA* deletion mutant was checked by PCR analysis and DNA sequencing.

### 2.4. Insertion of *undA* into pJN105 expression vector

The *undA*-EcoRI-F/*undA*-XbaI-R primers were used to amplify *undA* gene. The amplified fragment (856 bp) and the pJN05 vector were digested with *EcoRI* and *XbaI* to generate cohesive ends. Then, *undA* gene was ligated into the pJN105 plasmid (Newman and Fuqua, 1999), under the L-arabinose-inducible promoter control. The resulting plasmid was checked by sequencing.

### 2.5. MFE01 transformation by electroporation

Fresh colonies of MFE01 or mutants were resuspended in cold sterile 300 mM sucrose solution (Fisher Chemicals), washed two times, and resuspended in 100 μL of 300 mM sucrose. These competent cells and 150 ng of plasmid were added in 1 mm gap electroporation cuvettes (Fisher brand), electroporated at 1.8 kV for 5 ms, 200 Ω resistance and 25 μF capacitance. LB was added and mixtures were incubated for 1 h 30 min at 28°C with shaking. Transformed bacteria were plating on gentamycin supplemented LB agar.

### 2.6. Anti-*Legionella* activity tests

*L. pneumophila* inhibition by MFE01 or mutants was tested using a 6-well plate qualitative long-range inhibition assay as previously described by Corre et al. (2019). Three independents assays were performed for each condition.

### 2.7. HS-SPME/ GC-MS

VOCs were analyzed as previously described (Corre et al., 2021) with minor modifications. Briefly, an overnight culture of bacteria was inoculated (40 μL adjusted at OD_580_ = 1) in sterile headspace screw-cap vial (20 mL) and containing 10 mL of inclined LB agar. Vials were incubated for 24 h at 28°C to allow bacterial growth. Prior to analysis, the 100 μm SPME polydimethylsiloxane (PDMS) fiber (Shimadzu) was preconditioned in the bake-out oven of the injector as recommended by the manufacturer (250°C for 30 min). Extraction duration profiles were carried out for 30 min at 37°C under agitation (250 rpm). Desorption time was set at 5 min in the GC injection port sets at 250°C. GC-MS analyses were performed in a gas chromatograph GC-2010 Plus (Shimadzu). Helium was used as the carrier gas at a constant flow rate of 1.2 mL/min. The injector operated in the spitless mode and its temperature was set at 250°C. The separation of volatile compounds was performed on a SH-Rxi-5 ms column (30 m × 0.25 mmID, 0.25 mm; Shimadzu). The oven temperature program started at 40°C (held for 5 min), was raised at a rate of 4°C/min to 140°C (held for 2 min), raised at a rate of 6°C/min to 190°C (held for 2 min), and then raised at a rate of 15°C/min to 230°C (held for 1 min). The detection was performed by a QP2010 SE mass spectrometer sets in positive electron impact mode (EI) with 70 eV of electron energy. The electron multiplier was set by the auto-tune procedure. MS data were collected in a full scan mode over the *m*/*z* range from 35 to 400 (0.3 s/scan) Transfer line temperature was set at 230°C. At least three independent samples were analyzed for each condition. Once the raw data had been acquired, the peaks were automatically integrated on the basis of the area. Volatile compounds were identified using the NIST20 spectral library, based on the mass spectrum with the highest similarity score (minimum 87%). This is achieved by comparing the mass fingerprints of the unknown compounds eluted with those of 300,000 references. The identity of 1-undecene has already been confirmed by the use of an analytical standard in one of our previous studies (Corre et al., 2021). Only compounds systematically detected in all wild type samples were reported.

### 2.8. Biofilm culture and staining

Biofilm were developed in 24-well microplates with glass bottoms (Sensoplate, Greiner Bio-One). Overnight culture of MFE01 or mutants were adjusted at OD_580_ = 1 in LB and wells were filled with 1 mL of bacterial suspension. Plates were incubated at 28°C (*P. fluorescens*) or 37°C (*P. aeruginosa*) for 48 h and the medium was renewed after 24 h of incubation. When needed, biofilms were developed in separated plates to avoid volatile compounds interferences between cultures. The effect of QsdA on MFE01 biofilm structure was studied by adding 24 μg (i.e., 6.74 10–4 μmol) of QsdA to MFE01 cultures. After incubation, biofilms were washed twice with saline water (NaCl 9 g L^−1^) and stained with 5 μM Syto9 green-fluorescent dye (Invitrogen) for 15 min at room temperature. Five independent assays were performed for each condition.

### 2.9. Adhesion assay

Bacterial cultures were centrifuged 3 min at 8000 g and pellets were resuspended in saline water. Bacterial suspensions were then equilibrated at OD_580_ = 0.1 and 1 mL was deposited in a well of a 24-well polystyrene plate (Thermo Fisher Scientific). A 10 mm diameter sterile coverslip (Epredia) was deposited on the bottom of each well. After 2 h of incubation at 28°C in separate plates to avoid interferences by volatile compounds, each well was washed once with saline water and cells were stained with 5 μM Syto9 green-fluorescent dye for 15 min at room temperature. After one additional wash with saline water, coverslip was positioned between slide (VWR) and cover-slide (VWR) using ProlongDiamond^®^ (Invitrogen) as mounting medium. Three independent assays were performed for each condition.

### 2.10. Biofilm exposition to volatile compounds

To expose biofilm to MFE01 or mutants’ volatile compounds, two wells of a 24-well glass bottom plate were filled with 2 mL of LB agar (LBg). Then, 10 μL of a bacterial suspension equilibrated at OD_580_ = 10 were deposited on LBg and plates were incubated overnight prior to start biofilm culture. Each biofilm culture was adjacent to two LBg-filled wells. To expose biofilm to pure 1-undecene, the same protocol was used with modifications. A mixed cellulose membrane (Merk) was deposited on each LBg-filled well. A 220 μM 1-undecene solution in 100% ethanol was prepared from pure 1-undecene. 2 μL of this solution were deposited on membranes at the beginning of biofilm cultures. After 24 h of incubation, 1-undecene was renewed.

### 2.11. Confocal laser scanning microscopy

Biofilm observation and quantification of average thickness and biovolume were performed as described by Bourigault et al. (2021) using the Comstat 2.0 software (Heydorn et al., 2000). Surface coverage was determined as described by Cambronel et al. (2019) using the ImageJ software (Schneider et al., 2012).

### 2.12. Supernatant protein extraction

Overnight cultures (5 mL) were centrifuged at 7,500 g for 5 min at room temperature. 2 mL of supernatant were filtered through a 0.22 μm Millipore membrane (Merck). Trichloroacetic acid was added to 10% final concentration and mixture was incubated at 4°C overnight. Solution was then centrifuged at 13,000 g for 30 min at 4°C and supernatant was discarded. The pellet was washed twice with 2 mL cold 100% acetone (VWR chemicals) and centrifuged at 13,000 g, for 30 min at 4°C. Finally, the pellet was air dried for 30 min and resuspended with 20 μL of 2X Laemmli buffer (Nupage^®^, Invitrogen) containing 5% β-mercapto-ethanol. Three independent assays were performed for each condition.

### 2.13. SDS-PAGE analysis

Protein solutions were incubated for 5 min at 95°C and proteins corresponding to 1 mL of supernatant were separated on a 12% bis-acrylamide (Biorad) gel. Protein electrophoresis and visualization were performed as previously described (Bouteiller et al., 2020). Hcp identification by MALDI-TOF was carried out as previously described (Decoin et al., 2014).

### 2.14. Killing assay, mucoid phenotype and swimming motility

Killing assays, mucoid and swimming phenotypes were performed as previously described using *tssC* mutant strain, defective for T6SS activity, as negative control (Maurhofer et al., 1998; Decoin et al., 2014, 2015; Chane et al., 2019; Bouteiller et al., 2020). Three independent assays were performed for each condition.

## 3. Results

### 3.1. The *undA* deletion impairs 1-undecene emission and *Legionella pneumophila* aerial inhibition

**Figure 1:**
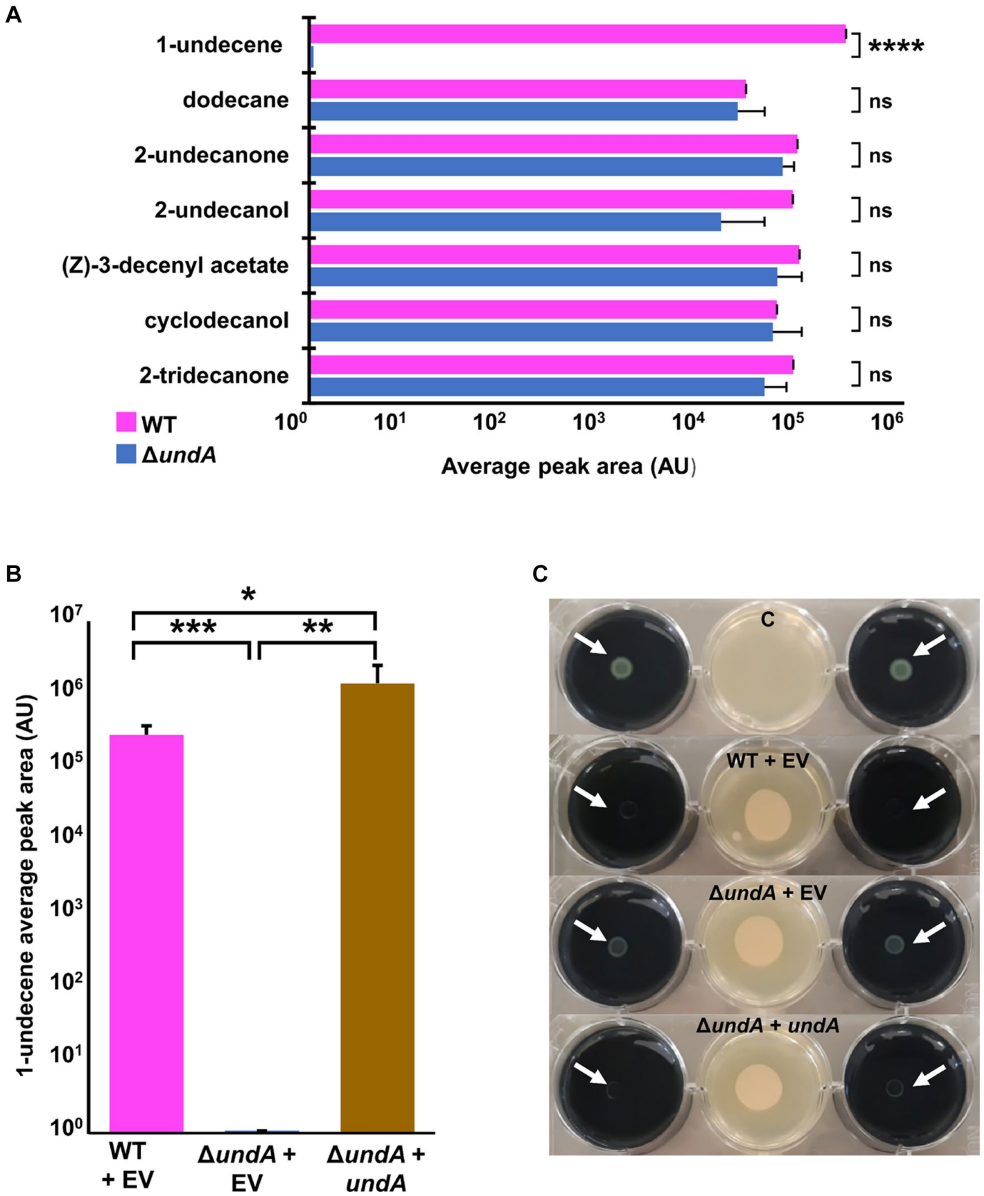
Emission of VOCs and *L. pneumophila* long-range inhibition. **(A)** Detection of main VOCs emitted from *P. fluorescens* MFE01 (WT) and its Δ*undA* mutant was realized by headspace SPME/GC-MS. Each strain was cultured in LB medium for 24 h and a suspension diluted at OD_580_ = 1 was deposited on a sterile GC vial filled with LB. Vials were then incubated for 24 h at 28°C and analyzed by SPME/GC–MS. VOCs detected from the Δ*undA* mutant headspace were compared to the VOCs systematically detected from the WT strain headspace. **(B)** Detection of 1-undecene emission from *P. fluorescens* MFE01 and mutants was realized by headspace SPME/GC-MS. Each strain was cultured in LB medium for 24 h and a suspension diluted at OD_580_ = 1 was deposited on a sterile GC vial filled with LB agar supplemented with 0.2% L-arabinose. Vials were then incubated for 24 h at 28°C and analyzed by SPME/GC-MS. ns, not significant; ^*^p < 0.05, ^**^p < 0.01, ^***^p < 0.001, and ^****^p < 0.001 (*t*-test). Data represent the mean of at least 3 independent experiments. Error bars indicate standard deviations. A.U. means arbitrary units. GC spectrum is in Supplementary Figure S2. **(C)** Volatile interference between *P. fluorescens* MFE01 containing pJN105 empty vector (WT + EV), *undA* mutant containing pJN105 empty vector (Δ*undA* + EV) or *undA* mutant containing *undA* cloned into pJN105 (Δ*undA* + *undA*) and GFP-tagged *L. pneumophila* Lens growth was determined using the 6-well plate assay. Growth of *L. pneumophila* Lens was monitored after 96 h of incubation at 28°C in absence or in presence of MFE01 or its mutants spread on the center of a 6-well plate. The absence of *L. pneumophila* lens growth indicates a volatile-dependent inhibitory phenotype. C means control without MFE01 or its mutants, EV means empty pJN105 vector. Initial plating of *L. pneumophila* Lens suspension on the agar plate is indicated by white arrows. Images are representative of three independent experiments.

In order to investigate impacts of 1-undecene emission on the physiology of MFE01, we managed to construct a MFE01 mutant unable to emit 1-undecene. The UndA enzyme was previously described in the *P. fluorescens* Pf-5 strain to catalyze 1-undecene synthesis by oxidative decarboxylation of cytoplasmic lauric acid (Rui et al., 2014) (Supplementary Figure S1). The presence of a single copy of *undA* gene (GenBank accession number: OQ434583.1) in MFE01 genome led to the construction of a MFE01 *undA* in frame deletion mutant, named Δ*undA*. In LB medium the Δ*undA* mutant showed no difference in growth compared to the wild type (WT) strain (Supplementary Figure S2). By using HS-SPME/GC–MS analysis, we analyzed VOCs emitted by MFE01 (WT) (Supplementary Figure S3A) and the Δ*undA* mutant. We defined a “core volatilome” in the WT strain as the VOCs systematically detected in our conditions. VOCs emitted with lower reproducibility were not considered here. Seven VOCs composed this “core volatilome,” belonging to the alkene, alkane, methyl ketone and alcohol chemical classes (Figure 1A). In the Δ*undA* mutant, the 1-undecene was never detected whereas the other VOCs were emitted in a similar amount comparatively to the WT strain (Figure 1A). Detection of 1-undecene by HS-SPME/GC-MS demonstrated that *ΔundA* mutant containing the empty pJN105 vector (*ΔundA +* EV) was not able to emit 1-undecene, in contrast to the WT strain containing the empty pJN105 vector (WT + EV) (Figure 1B; Supplementary Figure S3). In order to confirm the role of *undA* in 1-undecene emission, we cloned the *undA* gene in the L-arabinose inducible pJN105 plasmid (Newman and Fuqua, 1999). *In trans* expression of the *undA* gene in *ΔundA* mutant (*ΔundA* + *undA*) restored 1-undecene emission (Figure 1B). These results demonstrated that 1-undecene synthesis in MFE01 only occurs via UndA in our conditions. Interestingly, the overexpression of *undA* in the *ΔundA* + *undA* strain lead to an increase of 1-undecene emission (approximately a 5 folds increase in arbitrary units A.U.) and a weak additional synthesis of 1-4-undecadiene, a C11:1,4 alkadiene (Supplementary Figures S3B, S4). Then, the Δ*undA* mutant was tested to gain insight into the role of 1-undecene in *L. pneumophila* growth inhibition. Compared to the WT + EV strain, the Δ*undA* + EV mutant was attenuated in its ability to inhibit the growth of *L. pneumophila* (Figure 1C). Moreover, overexpression of *undA* in the Δ*undA* mutant restored antagonizing capacity of MFE01, confirming that *undA* gene expression in MFE01, and consequently 1-undecene emission, is important for the growth inhibition of *L. pneumophila* at distance. Interestingly, it appeared that the growth of *L. pneumophila* was still weakly inhibited by the (VCs) emitted by the Δ*undA* + EV mutant comparatively to the control condition (Figure 1C).

### 3.2. The T6SS of MFE01 and related phenotypes are not regulated by 1-undecene

**Figure 2:**
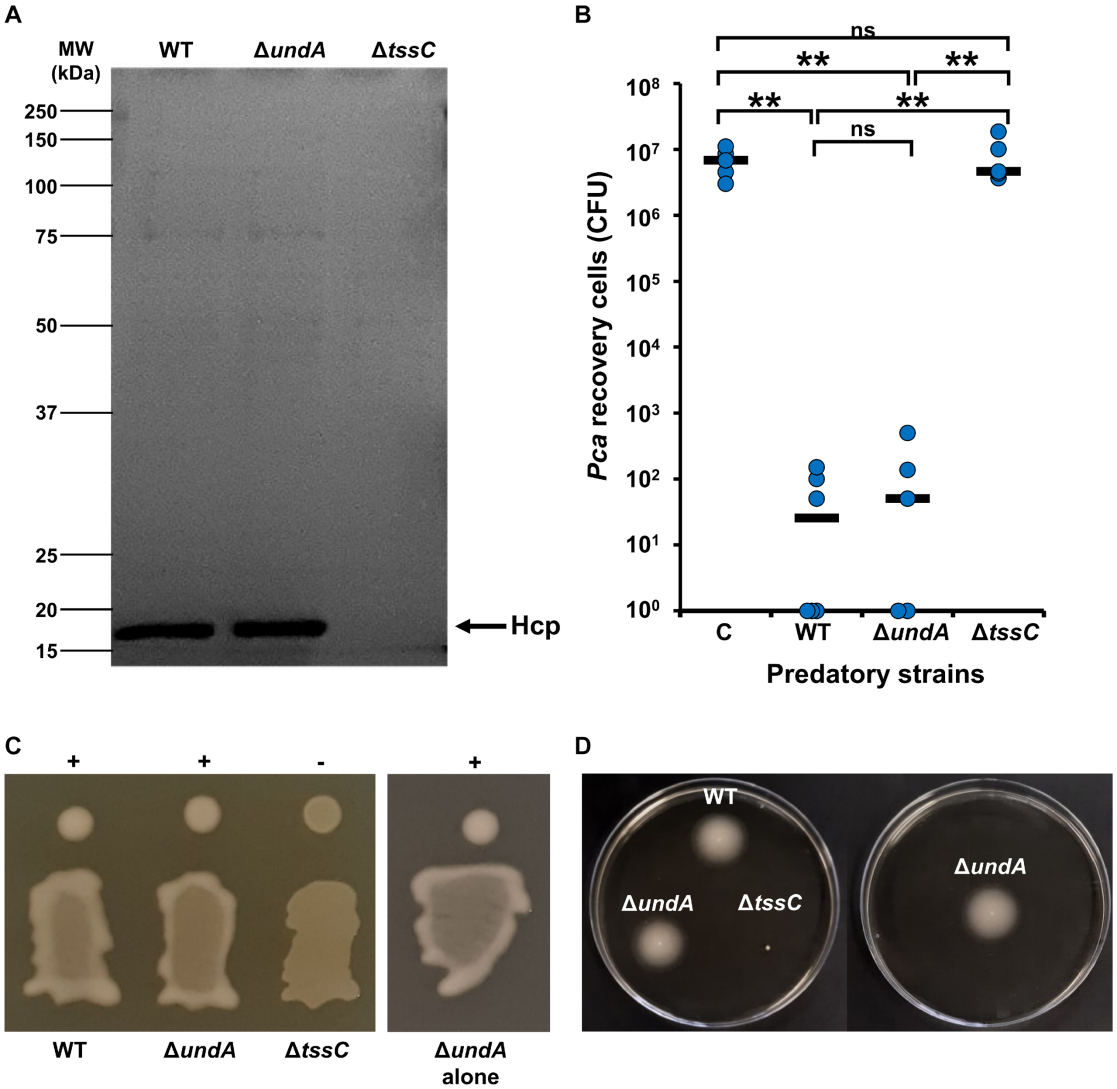
T6SS activity, mucoïdy and swimming motility. **(A)** Hcp secretion analysis. Supernatant of culture in stationary phase were concentrated and analyzed by SDS-PAGE (12% separation gel) and Coomassie staining. Arrows indicate the Hcp band, identified by Maldi/ToF. The image shown is representative of 3 assays (*n* = 3). **(B)** Killing activity of MFE01 and mutants. Contacts between the prey *P. atrosepticum* + PME6000: sfGFP-*mcherry* and the indicated predatory strains were performed. After 4 h of incubation at 28°C, number of recovered *Pca* were counted. ^**^p < 0.01; ns, not significant (*t*-test). C means control condition without predatory strain. WT: MFE01 Wild-type strain as predator. Δ*undA*: Δ*undA* strain as predator. Δ*tssC*: Δ*tssC* strain as predator. Horizontal bars represent median of 5 independent experiments. **(C)** Mucoidy was assessed on LB agar after 24 h of incubation at 28°C. +: mucoid. −: mucoid. Mucoidy of Δ*undA* mutant was performed in the same plate that the WT strain and alone. **(D)** Swimming assays were performed on 0.3% LB agar for 24 h at 28°C.

In previous work, we identified a potential link between 1-undecene emission and T6SS activity in a transposition mutant, but we were unable to explain this crosstalk (Corre et al., 2021). Moreover, previous studies demonstrated that T6SS is essential for antibacterial activity, swimming and mucoid phenotype in MFE01 (Decoin et al., 2014, 2015; Gallique et al., 2017b; Bouteiller et al., 2020). Then, we compared the Δ*undA* mutant with the WT strain and the MFE01 Δ*tssC* mutant (T6SS-inactivated mutant) (Decoin et al., 2015) for Hcp-secretion, antibacterial activity, mucoidy and swimming (Figures 2A–D, respectively). The secretion of Hcp proteins into the medium is described as the “hallmark” of a functional T6SS (Pukatzki et al., 2009). SDS-PAGE analysis of Δ*undA* mutant supernatant proteins revealed an Hcp secretion equivalent to that observed for the WT strain, corresponding to an active T6SS (Figure 2A). To confirm these results, we managed MFE01 or Δ*undA* killing assays against *P. atrosepticum* (*Pca*) (Decoin et al., 2014). After 4 h of contact, MFE01 and Δ*undA* killed in the same efficiency *P. atrosepticum in vitro*, decreasing cell counts by five-log compared to *Pca* counting without predatory cells (Figure 2B). During this experiment, each killing assay was performed in separate plates to avoid VCs interference between strains. Mucoid and swimming phenotypes were impaired in the T6SS-inactivated Δ*tssC* mutant while the Δ*undA* mutant was not impacted in its mucoid and swimming phenotype, whether in the same box or in a separate box from the WT strain (Figures 2C,D). These results indicated that 1-undecene does not regulate T6SS activity and related phenotypes.

### 3.3. The *undA* gene expression modulates biofilm maturation in *Pseudomonas fluorescens* MFE01

**Figure 3:**
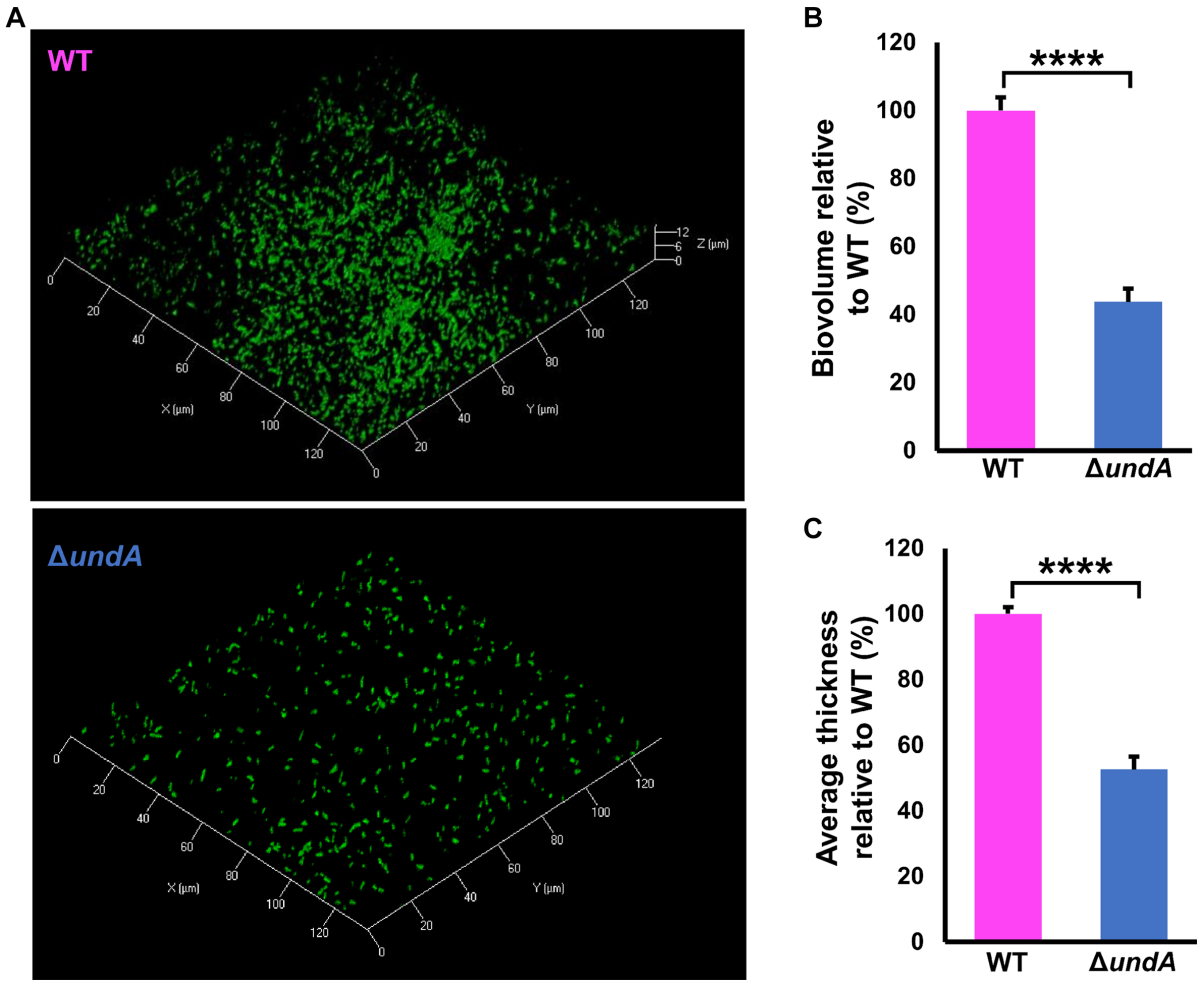
1-undecene emission and biofilm maturation. Biofilms were grown in static condition on a glass surface of a 24 well plate for 48 h at 28 °C in LB medium containing 0.2% L-arabinose. **(A)** Confocal laser scanning microscopy analysis of MFE01 + pJN105, Δ*undA* + EV and Δ*undA* + *undA* mutants. Representative biofilm 3D shadow representations are shown. Bacteria were visualized with the Syto 9^®^ green fluorescent nucleic acid stain. **(B)** Comstat analyses of biofilms biovolume. **(C)** Comstat analyses of biofilm average thickness. ^*^p < 0.05 and ^**^p < 0.01; ns, not significant (*t*-test). Data represent the mean of 5 independent experiments. Error bars represent standard error of the mean. EV means empty pJN105 vector.

**Figure 4:**
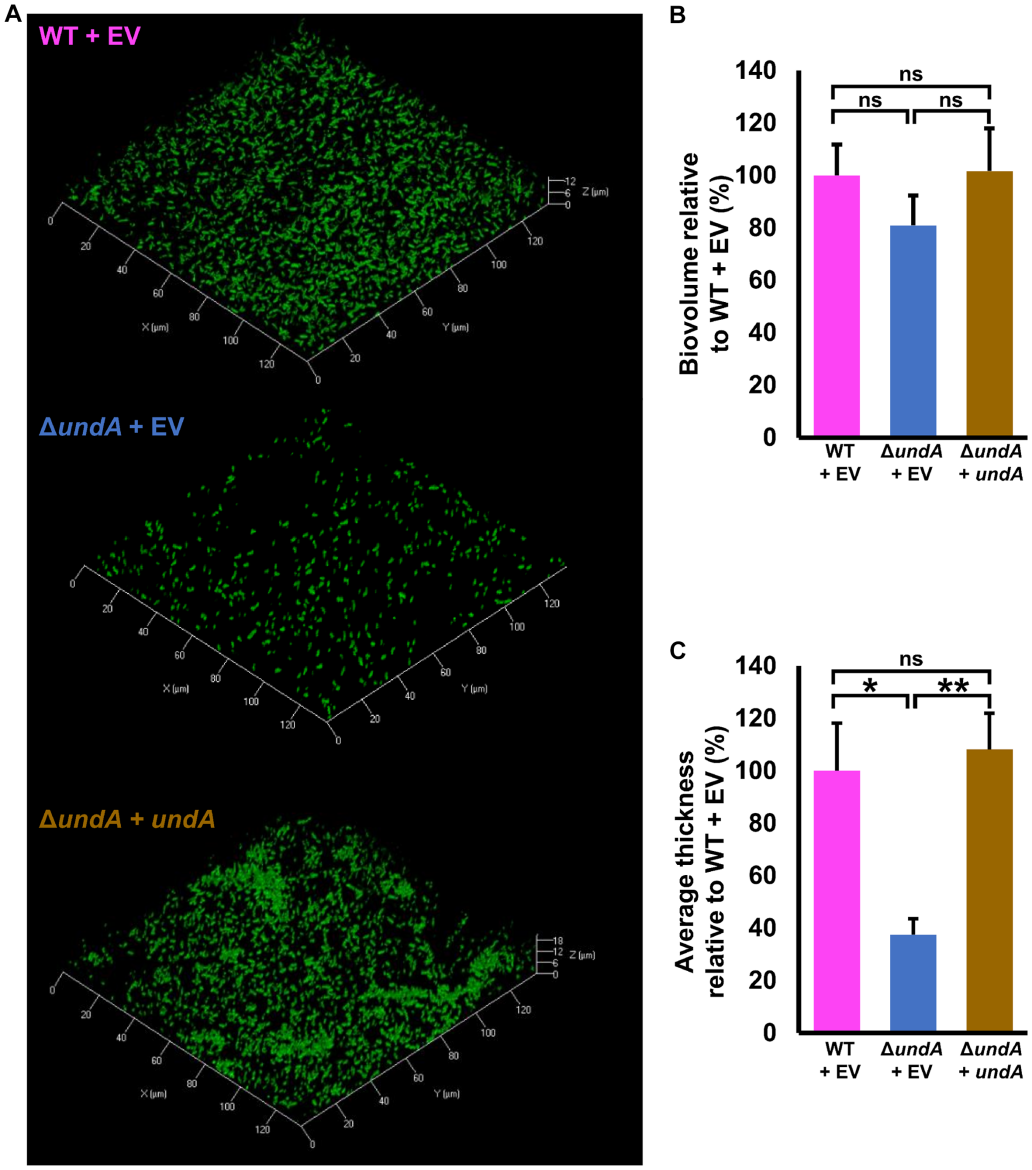
Effect of *undA* mutation on biofilm maturation. Biofilms were grown in static condition on glass surface of a 24 well plate for 48 h at 28°C in LB medium. **(A)** Confocal laser scanning microscopy analysis of MFE01 and its Δ*undA* deletion mutant. Representative biofilm 3D shadow representations are shown. **(B)** Comstat analyses of biofilms biovolume **(C)** Comstat analyses of biofilm average thickness. ^****^p < 0.0001; ns, not significant; (*t*-test). Data represent the mean of 5 independent experiments. Error bars indicate standard error of the mean.

To test 1-undecene impact on MFE01 coordinated phenotypes, we investigated biofilm formation and maturation in MFE01 and *ΔundA* mutant (Figure 3), and in the presence of the empty vector pJN105 or the *undA* gene cloned in the pJN105 vector (Figure 4). Biofilm formation assays were carried out on glass surface. To avoid VCs interferences, each biofilm assay was performed in separate compartment in the same incubator. Biofilms were visualized using confocal laser scanning microscopy (CLSM) after Syto9 nucleic acid-staining. The WT strain (Figure 3A) formed an aerial biofilm with cells dispersed in the matrix. These biofilms were heterogenous, containing aggregate in no mushroom-like structure and flatter zones. In contrast, the Δ*undA* mutant formed a flatter and non-matured biofilm, without notable cell aggregate. Biovolumes and average thicknesses were quantified by Comstat 2.0 analysis and corroborated these observations with a 60% and 50% decrease for each parameter, respectively (Figures 3B,C). Moreover, we evaluated the surface adhesion capacity of both WT and Δ*undA* strains. The two strains adhered on the surface equivalently, with an average surface covered corresponding to 6% of the total surface area (Supplementary Figure S5). As a result, *undA* gene seems to be implicated in biofilm maturation process and not in the ability to adhere on glass surface. In the presence of the pJN105 plasmid, the WT strain produced heterogenous mature biofilms in static conditions and contained cell-aggregates without mushroom like structures, while the Δ*undA* mutant formed a flatter non-maturated biofilm (Figure 4A). In these assays, the medium was supplemented with 0.2% L-arabinose and 50 μg mL^−1^ gentamycin sulfate. *In trans* overexpression of *undA* in Δ*undA* mutant restored the biofilm maturation but biofilms contained more aggregates than the WT strain biofilms. These observations were correlated by quantification of biovolume and average thickness using Comstat 2.0. The *undA* mutation did not significantly affect the biofilm biovolume (Figure 4B) but significantly reduced the average thickness (60% decrease). Intriguingly, in these conditions (with L-arabinose), the biofilm biovolume was not impacted by the *undA* deletion, contrary to that observed for the plasmid-free strains (Figure 3). We measured the impact of L-arabinose and pJN105 plasmid on 1-undecene emission in WT using HS-SPME/GC-MS. The presence of L-arabinose or plasmid did not alter the amount of 1-undecene emitted by MFE01 (Supplementary Figure S6).

### 3.4. In our conditions, biofilm maturation seems not AHL dependent in MFE01

A previous work using biosensor strains to detect AHL signals indicated the absence of medium or long chain AHL production by MFE01 (Gallique et al., 2017b). Since AHL-based QS is considered a key factor for biofilm development for many bacterial species, we wanted to test if MFE01 produced AHL in our conditions. However, biosensors are limited in the range of detectable molecules, and other lactone-containing QS molecules might be produced by MFE01. To challenge this possibility, we performed *in silico* detection of putative QS system’s genes in MFE01 genome using the tblastn software on the Basic Local Alignment Search Tool (BLAST) (Altschul et al., 1990). We searched for genes encoding AHLs synthesis proteins. Comparison using tblastn with RhlI (AAC44037.1), LasI (WP_134630090.1), LuxI (AAP22376.1) and TraI (AAZ50473.1) protein sequences versus MFE01 translated genome did not provide any results, suggesting an absence of gene encoding AHLs synthesis protein in MFE01 genome. The same method was applied to identify AHLs receptors homologous to *P. aeruginosa* RhlR (WP_003119559.1), LasR (WP_003082999.1) and QscR (WP_003118960.1) or *Alivibrio fischeri* LuxR receptor (WP_011263745.1), *Agrobacterium tumefaciens* TraR receptor (WP_010974900.1) and *E. coli* SdiA receptor (WP_001152715.1). Each of these proteins matched with a putative protein encoded by a single gene in MFE01 genome with an identity percentage in a range from 19.18% (TraR) to 30.8% (QscR) (Supplementary Figure S7A). Analysis of the putative protein sequence with Interproscan software (Jones et al., 2014) revealed that the corresponding putative protein contains two domains. The N-terminal domain (amino acids 4 to 166) belongs to the “Transcription factor LuxR-like, autoinducer-binding domain superfamily (IPR036693)” and the C-terminal to the “Winged helix-like DNA-binding domain superfamily (IPR036388)” (Supplementary Figure S7B). Thus, it appeared that this gene may encode an autoinducer-binding-domain-containing protein, able to bind to AHLs and to bind to DNA after homodimerization. Interestingly, this putative protein is identical to a protein of *Pseudomonas moraviensis* (MBH3446738.1) (Supplementary Figure S7C).

**Figure 5:**
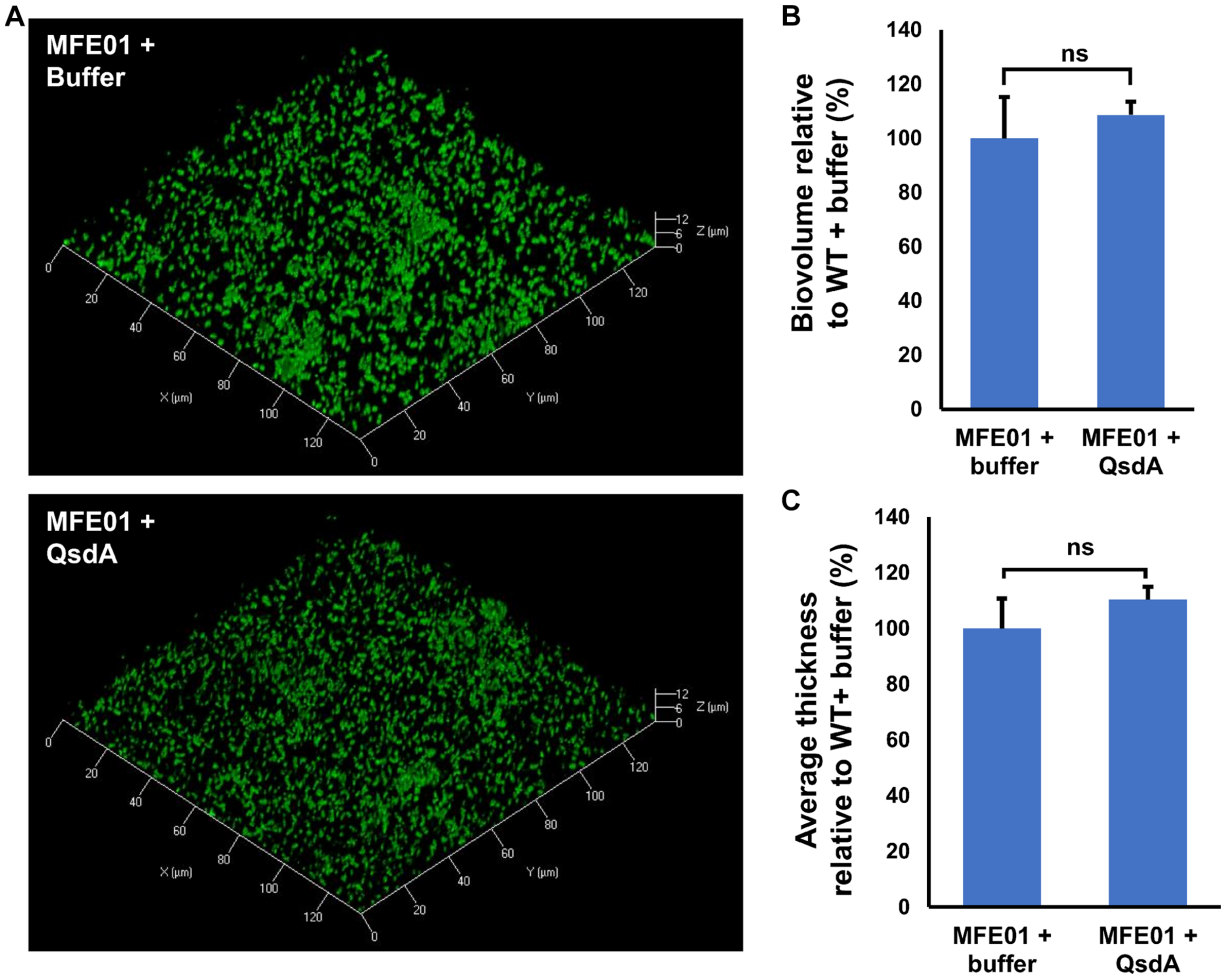
Effect of QsdA lactonase on MFE01 biofilm maturation. Biofilms were grown in static condition on a glass surface of a 24 well plate for 48 h at 28°C in LB medium. Medium was supplemented with 50 μL of buffer containing or not 24 μg of purified QsdA. **(A)** Confocal laser scanning microscopy analysis of MFE01 + Buffer or MFE01 + QsdA. Representative biofilm 3D shadow representations are shown. Bacteria were visualized with the Syto 9^®^ green fluorescent nucleic acid stain. **(B)** Comstat analyses of biofilms biovolume. **(C)** Comstat analyses of biofilm average thickness. ns, not significant; (*t*-test). Data represent the mean of 3 independent experiments. Error bars indicate standard error of the mean.

To determine if biofilm formation is AHLs-dependent in MFE01, under our conditions, i.e., in the absence of exogenous AHLs, we performed biofilm assays in presence of the lactonase QsdA. QsdA is an enzyme able to hydrolyze the lactone ring of a wide range of AHLs (Barbey et al., 2018), impairing biofilm formation of γ-proteobacteria using AHLs-based QS (Bourigault et al., 2021). No difference was observed on biofilm architecture, biovolume and average thickness by the QsdA addition to MFE01 cultures, suggesting that AHLs are not produced or do not influence MFE01 biofilm in our conditions (Figures 5A–C). On the contrary, *P. aeruginosa* H103 (AHLs producer) (Hancock and Carey, 1979; Tahrioui et al., 2019) exposure to QsdA caused a decrease in biofilm formation (Supplementary Figure S8).

### 3.5. 1-undecene is a potential aerial communication molecule

**Figure 6:**
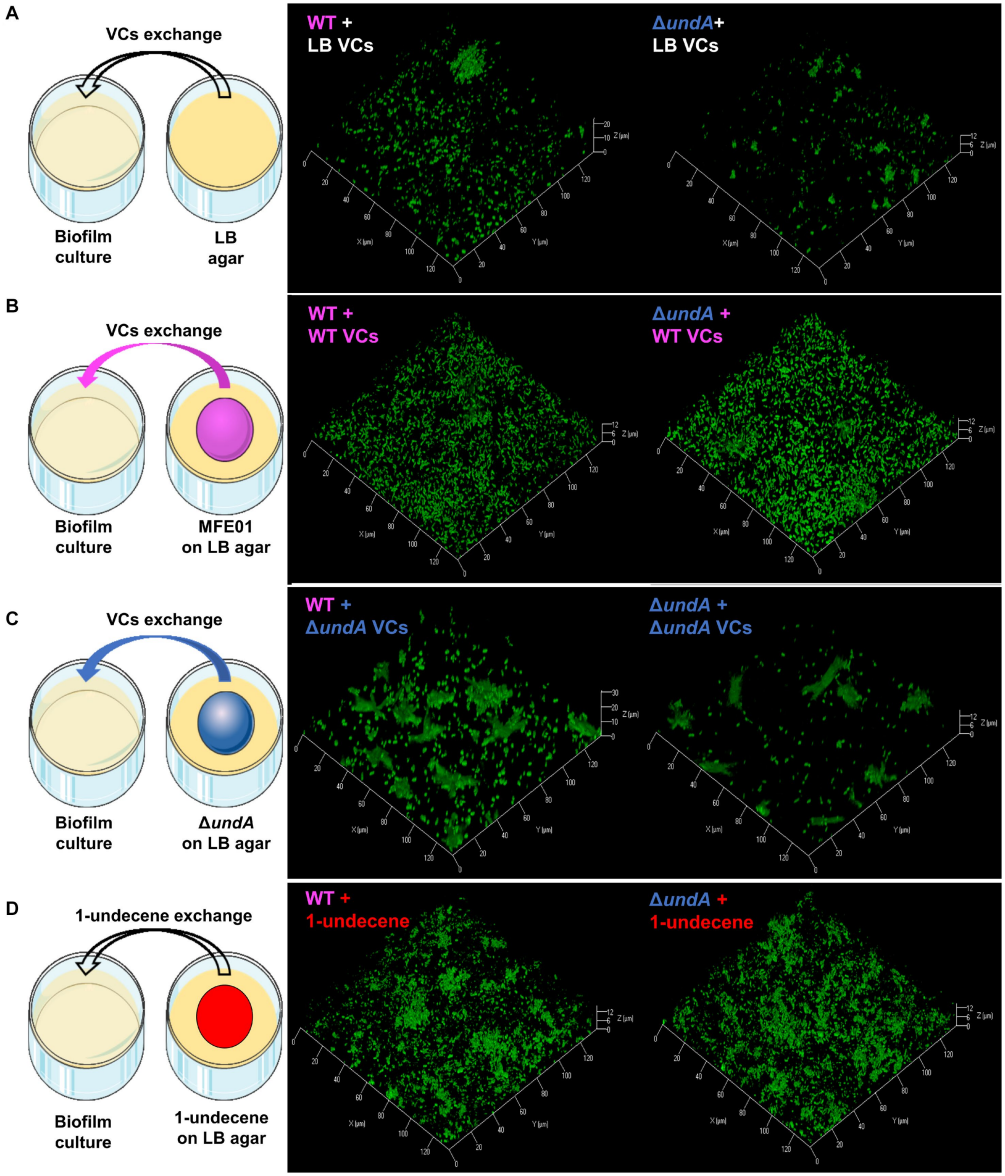
Biofilm maturation modulation by volatile compounds. **(A)** Exposition of biofilm to LB volatile compounds. **(B)** Exposition of biofilm to volatile compounds of MFE01 on LB. **(C)** Exposition of biofilm to volatile compounds of Δ*undA* mutant on LB. **(D)** Exposition of biofilm to pure 1-undecene. VCs means volatile compounds. The left panels correspond to schematic representations of experimental setups used for volatile compounds exposure, middle panels are confocal laser scanning microscopy analysis of MFE01 and right panels are confocal laser scanning microscopy analysis of Δ*undA* mutant exposed to the volatile compounds. Representative biofilm 3D shadow representations are shown. Biofilms were grown in static condition on a glass surface of a 24 well plate for 48 h at 28°C in LB medium. In each plate, 2 wells were filled with 2 mL of LB agar with or without 10 μL of MFE01 or Δ*undA* mutant culture at OD_580nm_ = 10 or 2 μL of 220 μM 1-undecene solution.

**Figure 7:**
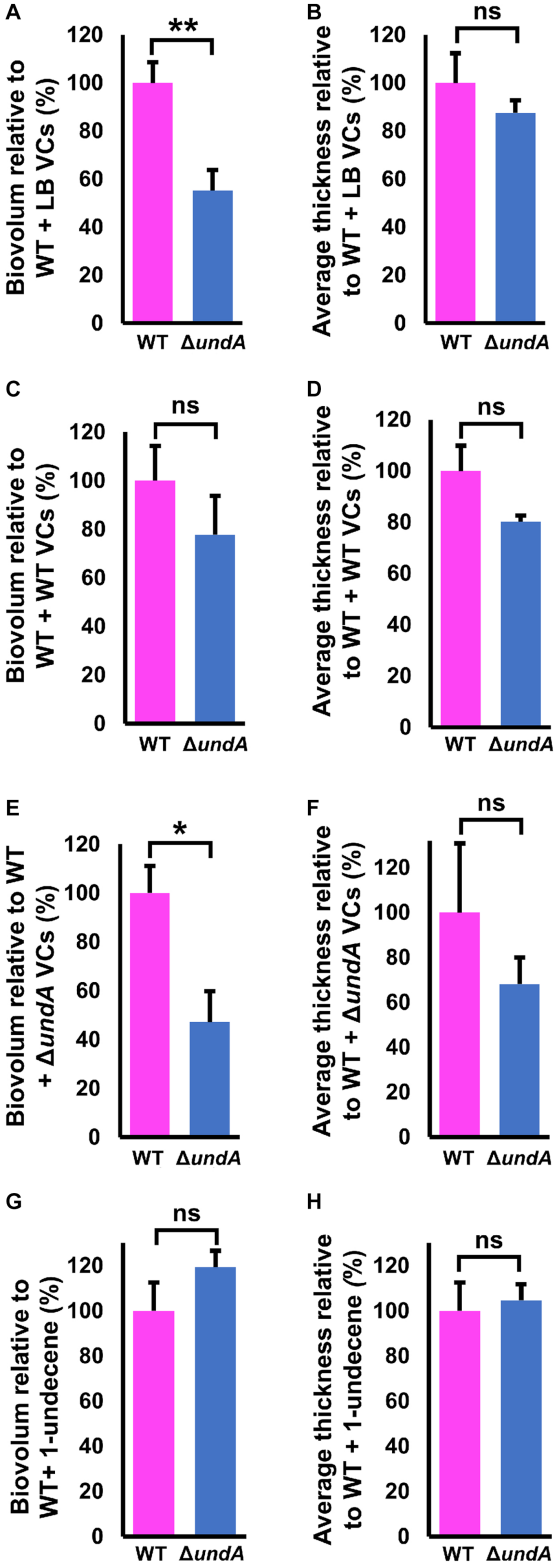
Quantitative analysis of biofilms developed after exposure to volatile compounds. **(A,B)** Comstat analysis of biofilms biovolume and average thickness of MFE01 and Δ*undA* mutant exposed to the LB volatile compounds. **(C,D)** Comstat analysis of biofilms biovolume and average thickness of MFE01 and Δ*undA* mutant exposed to the MFE01 volatile compounds. **(E,F)** Comstat analysis of biofilms biovolume (right panel) and average thickness of MFE01 and Δ*undA* mutant exposed to the Δ*undA* Volatile Compounds. **(G,H)** Comstat analysis of biofilms biovolume and average thickness of MFE01 and Δ*undA* mutant exposed to 2 μL of 220 μM 1-undecene solution, corresponding to 1-undecene quantity emitted by MFE01 in 24 h in LB medium. ^*^p < 0.05 and ^**^p < 0.01; ns, not significant (*t*-test). Data represent the mean of 5 independent experiments. Error bars indicate standard error of the mean.

To test whether 1-undecene might be an aerial communication molecule, we performed biofilm assays using a specific experimental setup (Figures 6A–D, left panels and Supplementary Figure S9). In each microtiter plate used for biofilm culture, two wells adjacent to the well exploited for biofilm development were filled with 2 mL of LB agar. Depending on the conditions, 10 μL of a bacterial suspension adjusted to OD_580_ = 10 of the WT or the Δ*undA* mutant or 2 μL of 220 μM 1-undecene solution were spotted on the LB agar. This setup allowed an exposure of the biofilm culture to the VCs emitted by the bacteria spotted on the LB agar or to pure 1-undecene. The quantity of pure 1-undecene used in this assay corresponds to 1-undecene measured in GC-vial after MFE01 growth during 24 h as described by Corre et al. (2021). The WT strain exposed to the VCs emitted by the LB (WT + LB VCs, Figure 6A, middle panel) formed a thick biofilm with mushroom-like aggregates while the Δ*undA* strain (Δ*undA* + LB VCs, Figure 6A, right panel) made a non-structured biofilm with numerous aggregates dispersed on the surface. The WT strain and the Δ*undA* mutant exposed to VCs emitted by the WT strain constituted homogenous biofilms with few non-mushroom aggregates, like the WT strain biofilm shown in Figures 3, 6B. In Figure 6C, MFE01 and Δ*undA* exposed to VCs emitted by the Δ*undA* mutant did not form similar biofilms. In this setup, the WT strain (Figure 6C, middle panel) developed a biofilm with some “hairy” microcolonies and surface adherent cells, whereas the Δ*undA* mutant formed a sparse biofilm with smaller number and size of microcolonies (Figure 6C, right panel). Exposed to pure 1-undecene, MFE01 and Δ*undA* formed biofilms that appear identical (Figure 6D) and similar to those visualized when *undA* was overexpressed (Figure 4C). Comstat 2.0 analysis (Figure 7) revealed that the *ΔundA* mutant (Figures 7A,B) built a biofilm reduced in its biovolume (50% decrease) without impact on the average thickness. In this experimental setup, biofilms seem to be slightly modified by the volatile compounds emitted by the LB agar medium in comparison with the biofilm developed by the WT strain showed in Figure 3. When exposed to MFE01’s VCs, the Comstat 2.0 analysis (Figures 7C,D) did not reveal any difference on biofilm biovolume or average thickness between the WT and Δ*undA* strains. Then it appeared that volatile compounds emitted by MFE01 restored the biofilm development of the Δ*undA* 1-undecene deficient strain. The Comstat 2.0 analysis during Δ*undA* mutant’s VCs exposition (Figures 7E,F) showed a decrease of biofilm biovolume and average thickness for the Δ*undA* mutant comparatively to the WT strain (50%). These observations confirmed that VCs, emitted by MFE01 and lacking in Δ*undA*, modulate biofilm maturation. No significant difference in biovolume or mean biofilm thickness was obtained after Comstat 2.0 analysis between WT and Δ*undA* mutant during 1-undecene exposure (Figures 7G,H). Thus, the presence of pure 1-undecene seems sufficient to restore the maturation of the biofilm.

## 4. Discussion

The UndA and UndB enzymes that catalyze 1-undecene synthesis by oxidative decarboxylation of lauric acid were previously studied by Rui et al. (2014, 2015). Currently, they are mainly studied in synthetic biology field for their high value in biofuel production, allowing valorization of multiple pollutants and wastes via various metabolic engineering strategies (Herman and Zhang, 2016; Chatterjee et al., 2018; Wang et al., 2018; Luo et al., 2019; Liu and Li, 2020; Salmela et al., 2020; Khanongnuch et al., 2022). Bioinformatical predictions designated UndB as a membrane bound desaturase (Rui et al., 2015). UndB is active on a broad range of free fatty acids (from C4:0 to C18:0), with a maximal affinity for lauric acid (C12:0) (Yunus et al., 2018, 2022). UndB allows emission of alkene in *Saccharomyces cerevisiae* which suggests UndB ability to translocate alkene across the membrane along with decarboxylation. In contrast the *undA* gene need co-expression of the human long-chain fatty acid transporter FATP1 to allow efficient 1-undecene emission in *S. cerevisiae* (Zhou et al., 2018). UndA is described as a cytoplasmic oxygen-activating nonheme iron-containing oxidoreductase. The reaction is probably catalyzed via substrate binding (i.e., free fatty acid from C10:0 to C14:0) to the Fe^2+^ center, which triggers electron transfer, leading to substrate oxidation, Fe^2+^ reduction and formation of H_2_O and CO_2_ (Rui et al., 2014). In *E. coli*, *undA* heterologous expression resulted in 1-undecene emission on LB medium (Rui et al., 2014) while medium supplementation in lauric acid is required for 1-undecene production during *undB* heterologous expression (Rui et al., 2015). These results indicate that in bacteria UndA decarboxylates fatty acid synthesized by cell metabolism while UndB decarboxylates fatty acids from the external environment. Our results confirmed the UndA requirement for 1-undecene emission by MFE01 and demonstrated that an overexpression of *undA* lead to an increase of 1-4-undecadiene emission. The role of UndA in 1-4-undecadiene synthesis was never stated but is consistent with UndA ability to bind to multiple substrates (Rui et al., 2014). Despite a low affinity, when UndA is present in large quantities, it could bind to dodec-4-enoic acid, allowing the synthesis of 1-4-undecadiene.

In this study, lack of 1-undecene emission decreased *L. pneumophila* aerial killing, even if slight inhibitory activity was still observed. We concluded that MFE01 may emit other VC able to limit *L. pneumophila* growth. Concordantly, in a previous work, one of the MFE01 transposition mutants was unable to inhibit the growth *L. pneumophila* (Corre et al., 2021). This mutant was impaired in emission of 1-undecene, 2-undecanone and 2-tridecanone. Purified 2-undecanone is described to possess nematocidal, antifungal and antibacterial activities (Gu et al., 2007; Popova et al., 2014; Giorgio et al., 2015; Lemfack et al., 2018), while 2-tridecanone has been associated with nematocidal activity, plant-bacteria communication and interference with bacterial social phenotypes (Ryu et al., 2004; Xu et al., 2015; Lemfack et al., 2018; López-Lara et al., 2018). Thus, it is probable that MFE01 inhibits *L. pneumophila* growth using a VCs cocktail, including mostly 1-undecene and likely 2-undecanone and/or 2-tridecanone.

Here, we demonstrated that the T6SS activity of MFE01 and related phenotypes, i.e., Hcp proteins secretion, killing activity, swimming and mucoidy, are not regulated by 1-undecene emission. These results thus invalidate our previous hypothesis formulated during the study of a transposition mutant impaired for VOCs emission and T6SS activity (Corre et al., 2021). This mutant contained an insertion of the transposon into one of the genes of tryptophan metabolism (the *trpE* gene). As a different phenotype was observed with an *in-frame* mutation in the *trpE* gene, we had also hypothesized a polar effect of the transposon insertion, resulting in an unexplained disturbance of a common regulatory pathway for the T6SS and the emission of VOCs. In literature, several studies report regulations of VOC emission and T6SSs by the two-component system (TCS) GacA/GacS (Cheng et al., 2013, 2016; Allsopp et al., 2017). Therefore, it seems relevant to study the impact of this TCS on MFE01 phenotypes. Experimentations concerning the role of the GacA/GacS system on 1-undecene emission and T6SS activity in MFE01 are underway in our laboratory.

One of our major findings is the key role of the *undA* expression, and therefore the 1-undecene synthesis, in the maturation of the MFE01 biofilm. This result is consistent with previously 1-undecene detection from *P. aeruginosa* biofilms (Koehler et al., 2020). Moreover, Rui and collaborators reported that *undA* gene is always associated with *rbdA* gene among various *Pseudomonas* genomes (Rui et al., 2014). RbdA seems to be implicated in signal recognition and biofilm modulation by modifying the ci-di-GMP homeostasis (An et al., 2010; Roy et al., 2012; Eilers et al., 2022). Work is planned to study the involvement of RbdA in maturation of biofilm during 1-undecene emission.

MFE01 genome analyses did not revealed any gene encoding AHLs synthesis protein and the addition of QsdA lactonase, purified from the quorum quenching agent *Rhodococcus erythropolis* R138, did not affect the maturation of biofilm in MFE01. It was previously demonstrated that QsdA both silences AHL communication from diverse bacteria and prevents biofilm formation in *Rhizobium rhizogenes* 5520^T^ (Barbey et al., 2018; Bourigault et al., 2021). These results are consistent with previously published infructive detection of AHL by biosensors (Gallique et al., 2017b), suggesting that MFE01 does not use AHLs-based QS system in our conditions. The lack of AHL-based QS and the key role of *undA* in biofilm maturation allow us to hypothesize that MFE01 may use 1-undecene as volatile communication molecule.

For this study, a novel experimental device was developed to analyze the effect of emitted volatile compounds on biofilm maturation. In this setup, the emission rate and the accumulation of VCs are continuous and biologically relevant. We think this device is more suitable for the study of aerial communication by VCs than other commonly performed experiments. In fact, several studies are based on the use of purified volatile compounds added at the outset of the experiment to evaluate their toxicity (Popova et al., 2014; De Vrieze et al., 2015; Lo Cantore et al., 2015; Corre et al., 2021). This may lead to a massive accumulation of volatile compounds in the first hours of experimentation followed by a dispersion. While these experiments are suited to demonstrate the toxicity of a VC, they do not seem to be appropriate for investigating the role of VCs in communication.

According to our experimental results, despite its hydrophobic part, 1-undecene remains sufficiently soluble in the aqueous phase of the biofilm to modify its maturation. This is coherent with VOCs detection in different sources of water (Chary and Fernandez-Alba, 2012) and the 1-undecene solubility in water of 0.3432 mg/L, corresponding to 2.22 μmol per liter, accordingly to the PubChem database.^2^ Another volatile communication molecule, the 3-hydroxypalmitic acid methyl ester (3-OH PAME), was previously described for its implication in gaseous and water phase communication (Clough et al., 1994; Flavier et al., 1997). This VOC is synthesized by the *Ralstonia solanacearum* PhcB enzyme and is detected by the PhcR sensor histidine kinase, leading to expression of genes involved in virulence towards *Solanum tuberosum* (Clough et al., 1994, 1997; Flavier et al., 1997; Schell, 2000). Schell (2000) proposed the Phc system as a confinement sensing system. In this pathosystem, the accumulation of 3-OH PAME produced by *R. solanacearum* is detected as a signal indicating a confined space that is a favorable niche to trigger virulence. Here we report that the environmental strain *P. fluorescens* MFE01 could use 1-undecene as an aerial communication signal. Indeed, the Δ*undA* mutant was not able to mature its biofilm, but the 1-undecene emitted by the wild strain or exposure to pure 1-undecene at biologically relevant concentration restored its biofilm maturation capacity. These finding suggest that during 1-undecene emission, by an unknown mechanism, MFE01 detects it, leading biofilm formation modulation. To our knowledge, this is the first description of 1-undecene as intraspecific bacterial aerial communication molecule. This leads us to propose 1-undecene as a member of the restrictive list of volatiles autoinducers, as well as 3-OH PAME (Clough et al., 1994, 1997; Flavier et al., 1997; Schell, 2000) and indole (Wang et al., 2001; Lee et al., 2007, 2009; Lee and Lee, 2010; DeJong et al., 2017). Then, it appears that volatile compounds, including 1-undecene, may act as indicator for a high cell density or confined space. Characterization of other volatile autoinducers and their detection systems seems to be essential for identifying the biological roles of VOCs-based communication. Interestingly, Prakash et al. (2021) have already demonstrated that 1-undecene plays a role in interspecific communication. Indeed, the nematode *Caenorhabditis elegans* can specifically detect the 1-undecene emitted by *P. aeruginosa*, leading to a flight and early triggering of the immune response. Taken together, these results suggest that 1-undecene may be used as a toolbox by *Pseudomonas*, during competition, virulence or inter- and intra-species communication.

Surprisingly, VCs emitted by the LB medium modulated MFE01 biofilm. This unexpected finding highlights MFE01 ability to integrate signal from various VCs. Another interesting result was the modification of biofilm maturation in presence of L-arabinose in the medium, despite the lack of effect on 1-undecene emission. This may be explained by the ability of *P. fluorescens* bacteria to catabolize arabinose (Lockwood and Nelson, 1946), and to integrate it in its biofilm matrix (Hung et al., 2005; Raza et al., 2012). Thereby, arabinose supplementation, used for activation of pJN105 promotor, may modify MFE01 metabolism and EPS matrix of biofilm, explaining the observed differences.

To conclude, *P. fluorescens* MFE01 seems to use its own main VOCs (i.e., 1-undecene) as an aerial communication molecule. We hypothesize that MFE01 may possess dedicated receptors for different VCs. This confirms that bacterial communication is a complex network of multiplex chemical signals detected by specific receptors. An environmental strain such as *P. fluorescens* MFE01, lacking AHL-based QS, appears to be a suitable model for studying undervalued modes of communication to date.

## Data availability statement

The datasets presented in this study can be found in online repositories. The names of the repository/repositories and accession number(s) can be found at: https://www.ncbi.nlm.nih.gov/genbank/, OQ434583.

## Author contributions

CD: Conceptualization, Data curation, Formal analysis, Investigation, Methodology, Visualization, Writing – original draft, Writing – review & editing. YB: Formal analysis, Investigation, Methodology, Writing – review & editing. TO: Formal analysis, Investigation, Methodology, Writing – review & editing. MN: Formal analysis, Investigation, Methodology, Writing – review & editing. CB: Investigation, Methodology, Writing – review & editing. XL: Funding acquisition, Writing – review & editing. Y-KG: Writing – review & editing. JV: Conceptualization, Formal analysis, Funding acquisition, Investigation, Methodology, Supervision, Writing – original draft, Writing – review & editing. AM: Conceptualization, Formal analysis, Funding acquisition, Investigation, Project administration, Supervision, Validation, Visualization, Writing – original draft, Writing – review & editing.

## Footnotes

^1^ https://tmcalculator.neb.com/#!/main

^2^ https://pubchem.ncbi.nlm.nih.gov/compound/1-Undecene
