# Supplementary material for: Pseudomonas fluorescens MFE01 uses 1-undecene as aerial communication molecule
Source: Front Microbiol. 2023 Oct 16;14:1264801. doi: 10.3389/fmicb.2023.1264801 (PMC10614000; doi:10.3389/fmicb.2023.1264801)

Supplementary Material

Pseudomonas fluorescens MFE01 uses 1-undecene as volatile signal for communication

Charly A. Dupont[1,2], Yvann Bourigault[1,2], Théo Osmond[1,2], Maëva Nier[3], Corinne Barbey[1,2], Xavier Latour[1,2], Yoan Konto-Ghiorghi[1,2], Julien Verdon[3] and Annabelle Merieau[1,2*]

*** Correspondence:**
annabelle.merieau@ univ-rouen.fr

Supplementary Figures and Tables

Supplementary Table 1. Strains and plasmids used in this study.

**Strains**	**Relevant Characteristics**	**Reference/Source**
Pseudomonas fluorescens		
MFE01	environmental isolate, Rif^R.	Decoin et al., 2014
MFE01 Δ*undA*	MFE01 with a *in frame* central deletion in *undA* gene.	This study
MFE01Δ*tssC*	MFE01 with a *in frame* central deletion in *tssC* gene.	Decoin et al., 2015
MFE01 + pJN105	MFE01 with pJN105 empty vector, Gm^R.	Bouteiller et al., 2020
MFE01 Δ*undA* + pJN105	MFE01 Δ*undA* with pJN105 empty vector, Gm^R.	This study
MFE01 Δ*undA* + *undA*	MFE01 Δ*undA* with pJN105 vector carrying wild-type *undA* gene, Gm^R.	This study
Pseudomonas aeruginosa		
H103	Wild-type strain; prototroph derivative of PAO1	Hancock and Carey, 1979
Legionella pneumophila		
CIP 108286	Virulent *L. pneumophila* serogroup 1, strain Lens, gfp, Cm^R.	Bigot et al., 2013
Pectobacterium atrosepticum		
CFBP6276	Potato soft rot pathogen.	Smadja et al., 2004a
Escherichia coli		
Top10®	F- *mcrA* Δ(*mrr-hsdRMS-mcrBC*) Φ80l*acZ*ΔM15 Δ*lac*X74 *recA1 araD139* Δ(*araleu*)7697 *galU galK rpsL* (Str^R) *endA1 nupG.*	ThermoFischer Scientific
S17.1	RP4-2-Tc::Mu, *aph*::Tn7, *recA*, Sm^R, donor strain for conjugation.	Simon et al, 1983
**Plasmids**		
pAKE604	Conjugative suicide vector, *oriT*, *lacZ*, *sacB*, Ap^R, Km^R.	El-Sayed et al., 2001
pAKE604:Δ*undA*	pAKE604 plasmid carrying the sequence used for *undA* gene *in-frame* deletion.	This study
pJN105	Arabinose-inducible cloning vector, derivative of pBBR1-MCS5, Gm^R.	Newman et al., 1999
pJN105 :*undA*	pJN105 plasmid carriyng the *undA* gene under control of an L-arabinose inducible promoter. Gm^R.	This study
pME6000	Cloning vector, derivative of pBBR1-MCS, Tc^R.	Maurhofer et al., 1998
pME6000 :*luxR*-P_{luxI} ::gfp_{asv}-*cfp*	pME6000-*cfp* plasmid with a P_{luxI}::gfp_{asv} transcriptional fusion under the control of *luxR* expression. Tc^R.	Chane et al., 2019

Supplementary Table 2. Primers used in this study

**Mutagenesis primers**	**Sequences (5' to 3')**
M1-undA	GGACATGGCTTGCTACATGG
M2QC-undA	ATACTTCGTGGGTCCAGGTGATGATCGGTTGAATGTGTCGATCAC
M3QC-undA	TCATCACCTGGACCCACGAAGTATACCGAAAGCTGATCGAACTG
M4-undA	ACTAAACTTACGCCAAACGTCC
**overexpression primers**	**Sequences (5' to 3')**
undA-EcoRI-F	AATAAGAATTCCAAAAGGAGCCGAACAGTGA
undA-XbaI-R	TAATAATCTAGAGCATGACCCACCGCTTTC

Supplementary Figure 1. Reaction of 1-undecene synthesis by UndA enzyme.
UndA enzyme catalyzes lauric acid oxidative decarboxylation using dioxygen. This reaction forms 1-undecene, carbon dioxide, water and reduces the iron center of UndA. (Reaction write accordingly to Rui et al., 2014)

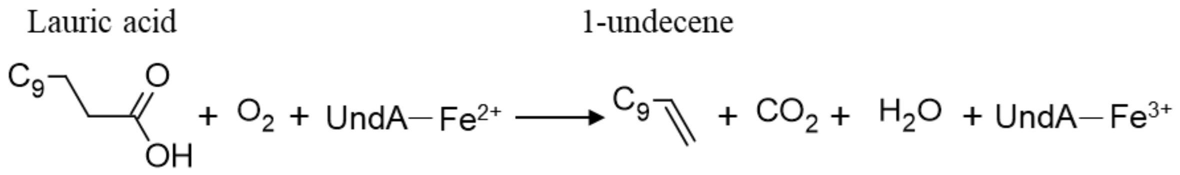

Supplementary Figure 2. Growth curves of MFE01 and *undA* mutant in LB medium

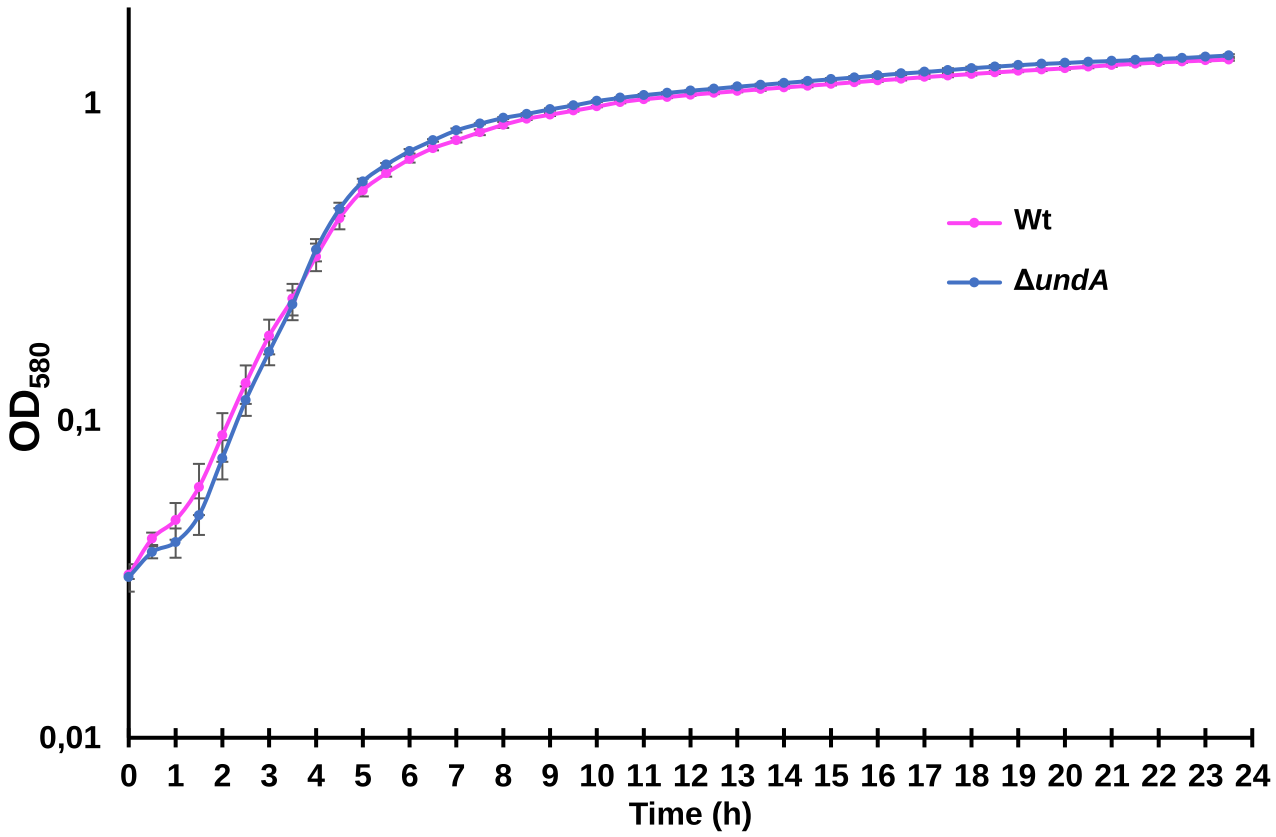

Supplementary Figure 3. Representative TIC chromatograms
(A) Representative TIC chromatogram of VOCs detected from *P. fluorescens* MFE01 WT strain. VOCs detected in LB alone were not considered and therefore only those present only in the presence of MFE01 were numbered. Their identity, after comparison of mass fingerprints with the NIST20 database (Best match with an identity score ≥ 87%) are listed: 1, 1-undecene. 2, dodecane. 3, undecanal. 4, 2-undecanone. 5, 2-undecanol.6, (Z)-3-decenyl acetate. 7, (E)-5-decenyl acetate. 8, cyclodecanol. 9, 2-tridecanone. 10, hexadecane. 11, ethanone, 1-cyclododecyl. 12, (Z)-9-tetradecenyl acetate. **(B)** Representative 1-undecene and 1-4 decadiene peaks obtained by overlaying the TIC chromatograms and focusing on the retention time of 16.85, which corresponds to that of 1-undecene.

A

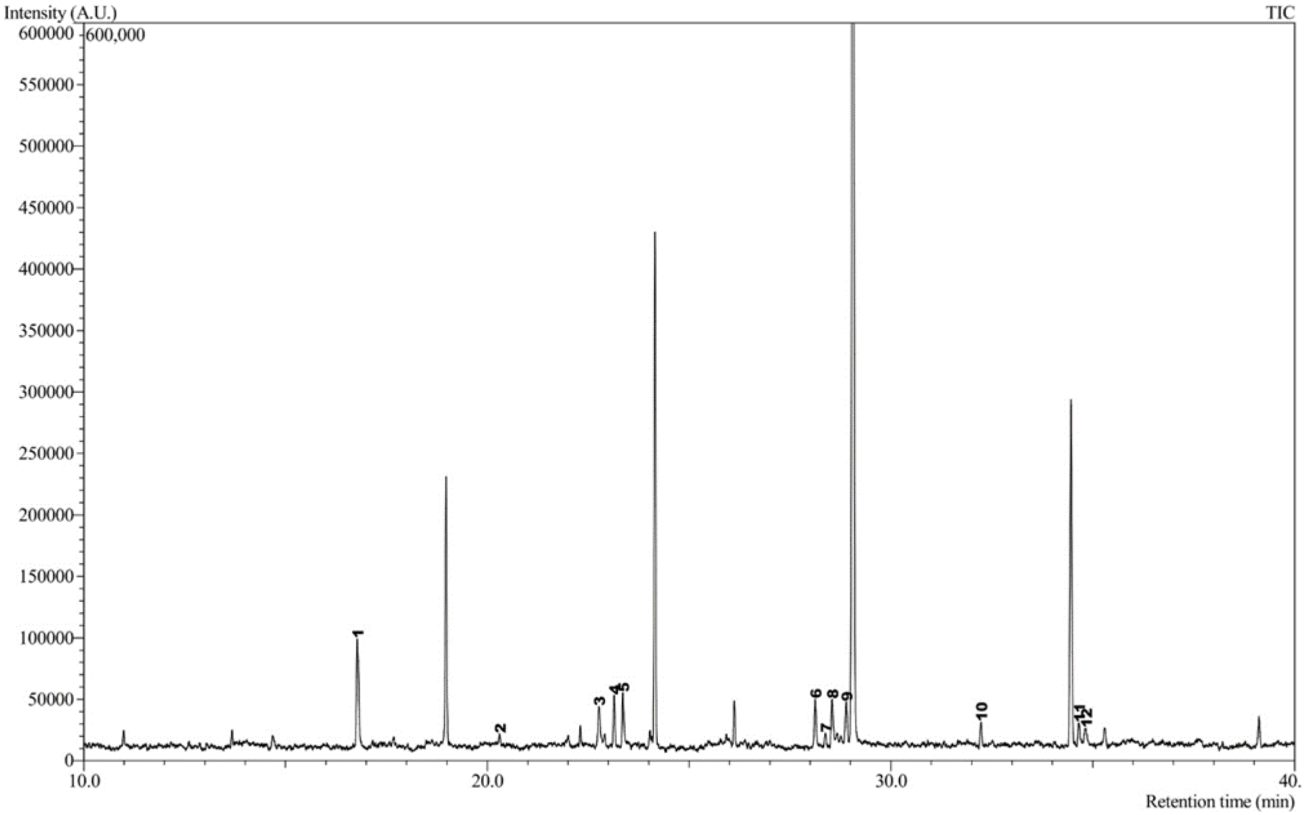

B

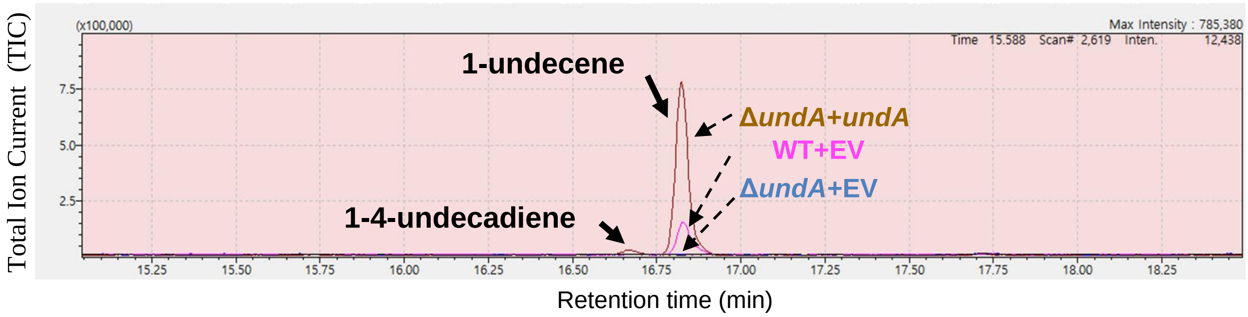

Supplementary Figure 4. 2D structures of 1-undecene and 1-4-undecadiene
Structure of **(A)** 1-undecene ($C_{11}H_{22}$; CAS 821-95-4) and **(B)** 1-4-undecadiene ($C_{11}H_{20}$; CAS 53786-93-9) were extracted from Pubchem database (https://pubchem.ncbi.nlm.nih.gov/)

A

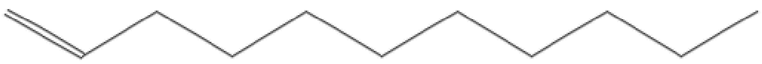

B

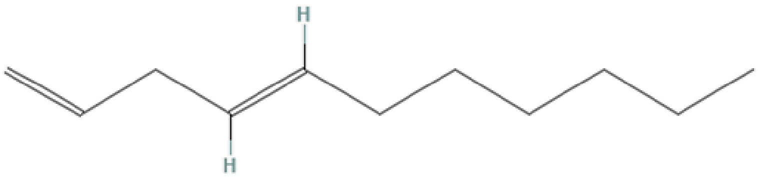

Supplementary Figure 5. Surface adhesion WT and Δ*undA* strains.
(A) Representative adhesion 2D top view of MFE01. **(B)** Representative adhesion 2D top view of Δ*undA* mutant. **(C)** Analysis of the total surface adhesion coverage. Adhesions were performed in static condition on glass surface for 2h at 28°C in saline water. Bacteria were stained with Syto9 and observed using CLSM. Data represent the mean of 3 independent experiments. Error bars indicate standard deviations. t-test. n.s: not significant.

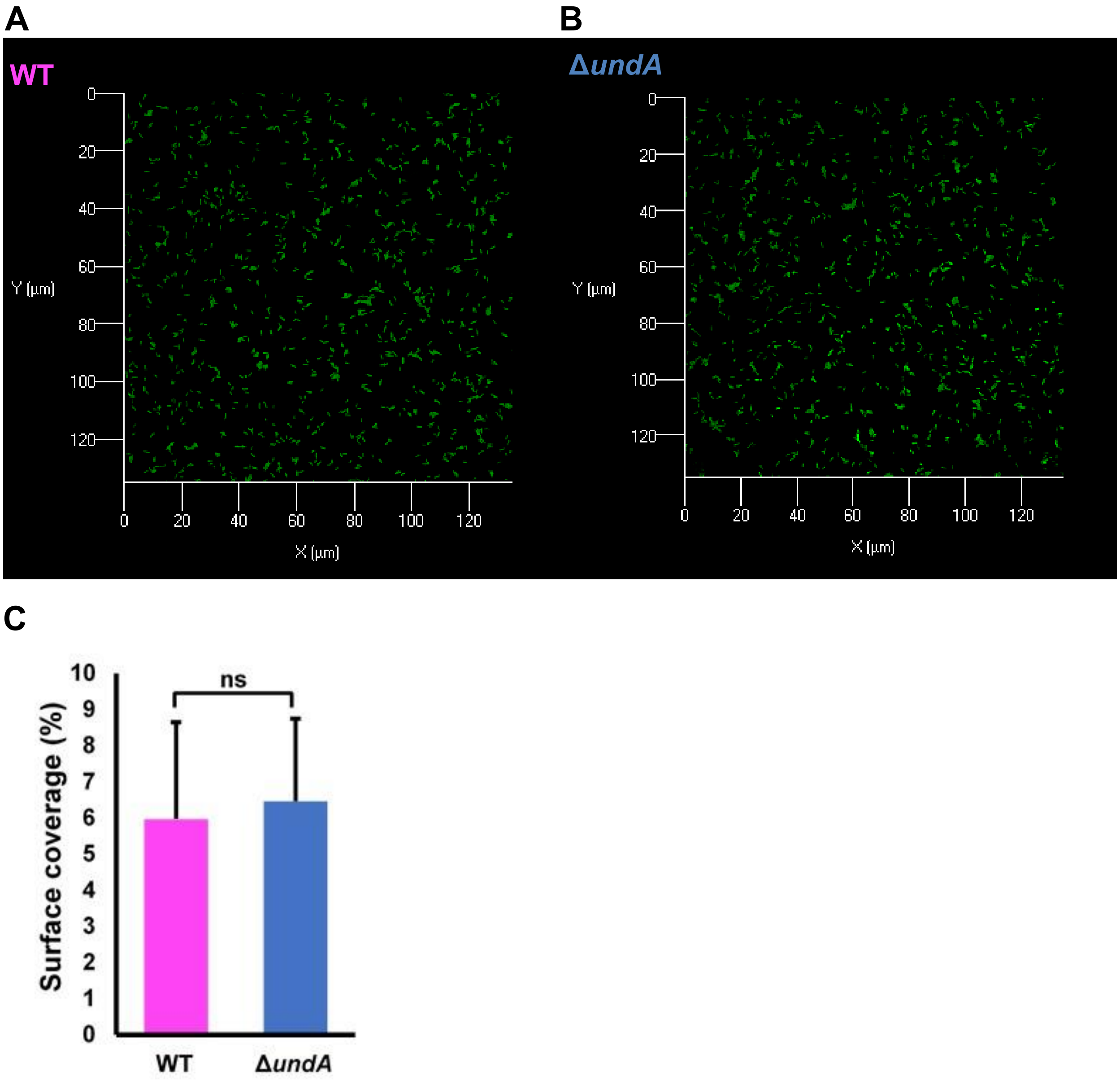

Supplementary Figure 6. Quantification of 1-undecene emitted by the WT strain in the different conditions used in this study. Detection of 1-undecene emission from *P. fluorescens* MFE01 transformed or not with pJN105 was performed by headspace SPME/GC-MS. Each strain was cultured in LB medium supplemented or not with 0.2% arabinose for 24 h and a suspension diluted at OD_{600} = 1 was deposited on a sterile GC vial filled with LB agar supplemented or not with 0.2% L-arabinose. Vials were then incubated for 24h at 28°C and analyzed by SPME/GC-MS.; n.s : not significant (t-test). Data represent the mean of 3 independent experiments. Error bars indicate standard deviations. A.U. indicates arbitrary units.

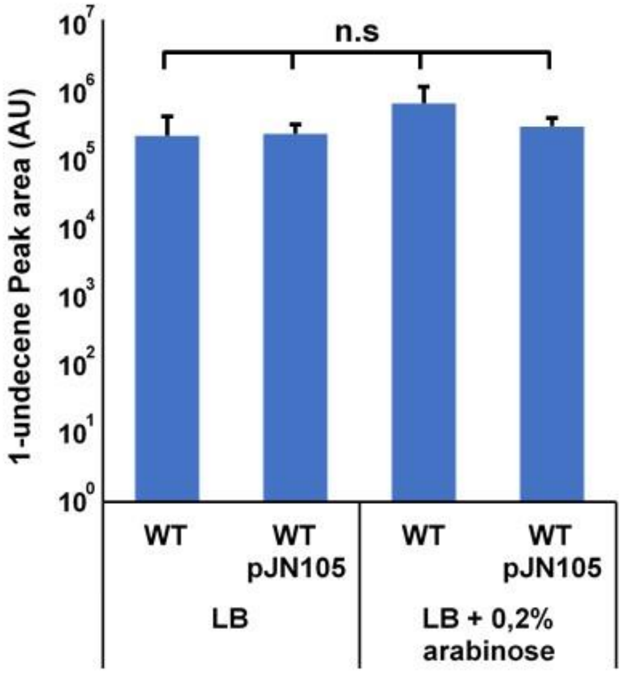

Supplementary Figure 7. *In silico* research of QS system's genes in *P. fluorescens* MFE01. **(A)** Amino acid sequence alignment between *N*-acyls homoserine lactones receptors identified in different γ-proteobacteria and the unique putative protein identified in MFE01 using tblastn software. **(B)** Schematic representation of bidimensional structure of the putative AHLs binding receptor of MFE01. **(C)** Amino acid sequence alignment between *P. moraviensis* and *P. fluorescens* MFE01 putative AHLs binding receptors.

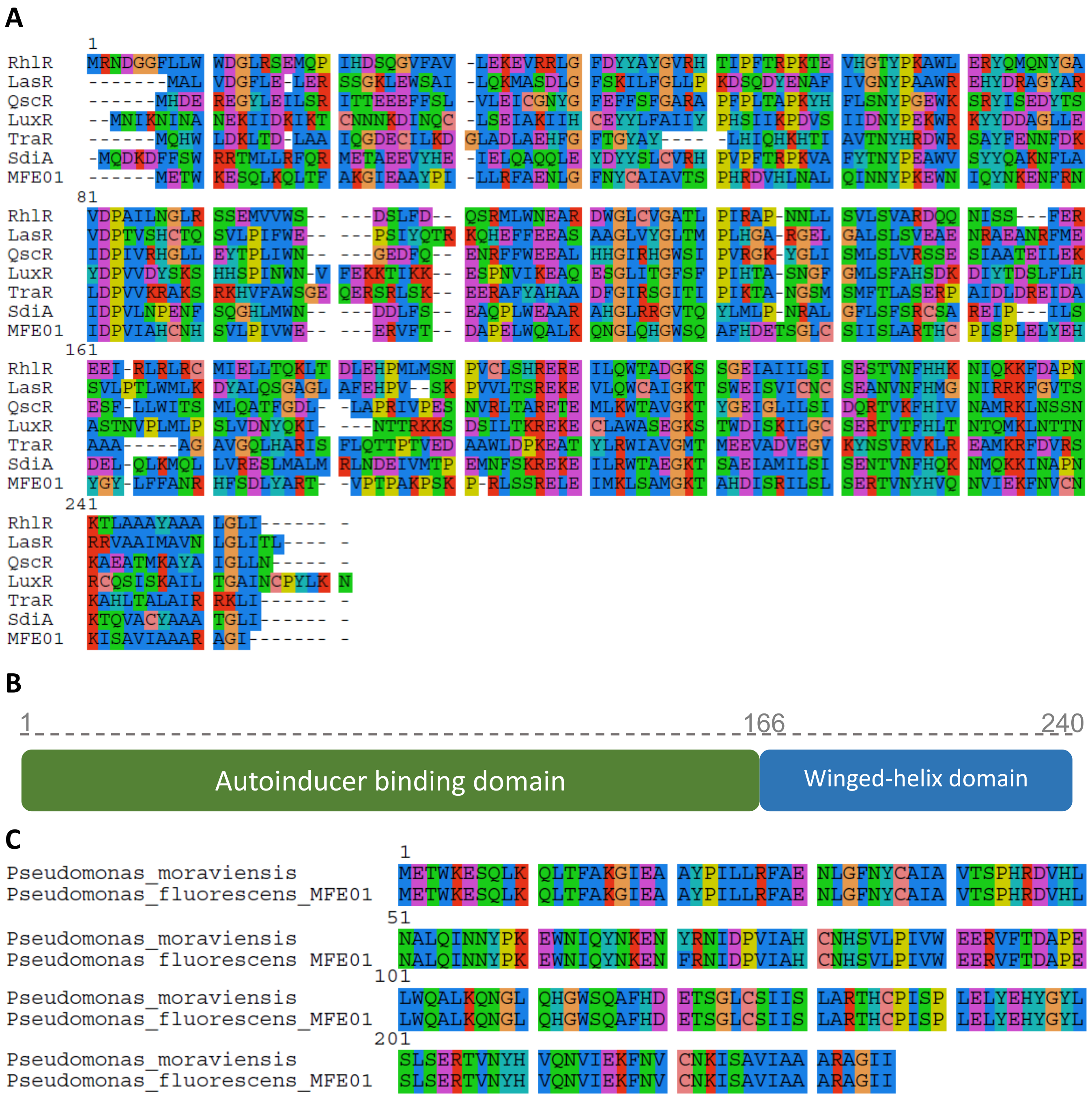

Supplementary Figure 8. Effect of QsdA lactonase on H103 biofilm maturation
Biofilms were grown in static condition on glass surface of a 24 well plate for 48h at 37°C in LB medium. Medium was supplemented with 50 µL of buffer containing or not 24 µg of purified QsdA. **(A)** Confocal laser scanning microscopy analysis of H103+Buffer (control) or H103+QsdA. Representative biofilm 3D shadow representations are shown. Bacteria were visualized with the Syto 9® green fluorescent nucleic acid stain. **(B)** Comstat analyses of biofilms biovolume. **(C)** Comstat analyses of biofilm average thickness. *, P<0,05; **,P<0,01 (t-test). Data represent the mean of 3 independent experiments. Error bars indicate standard error of the mean.

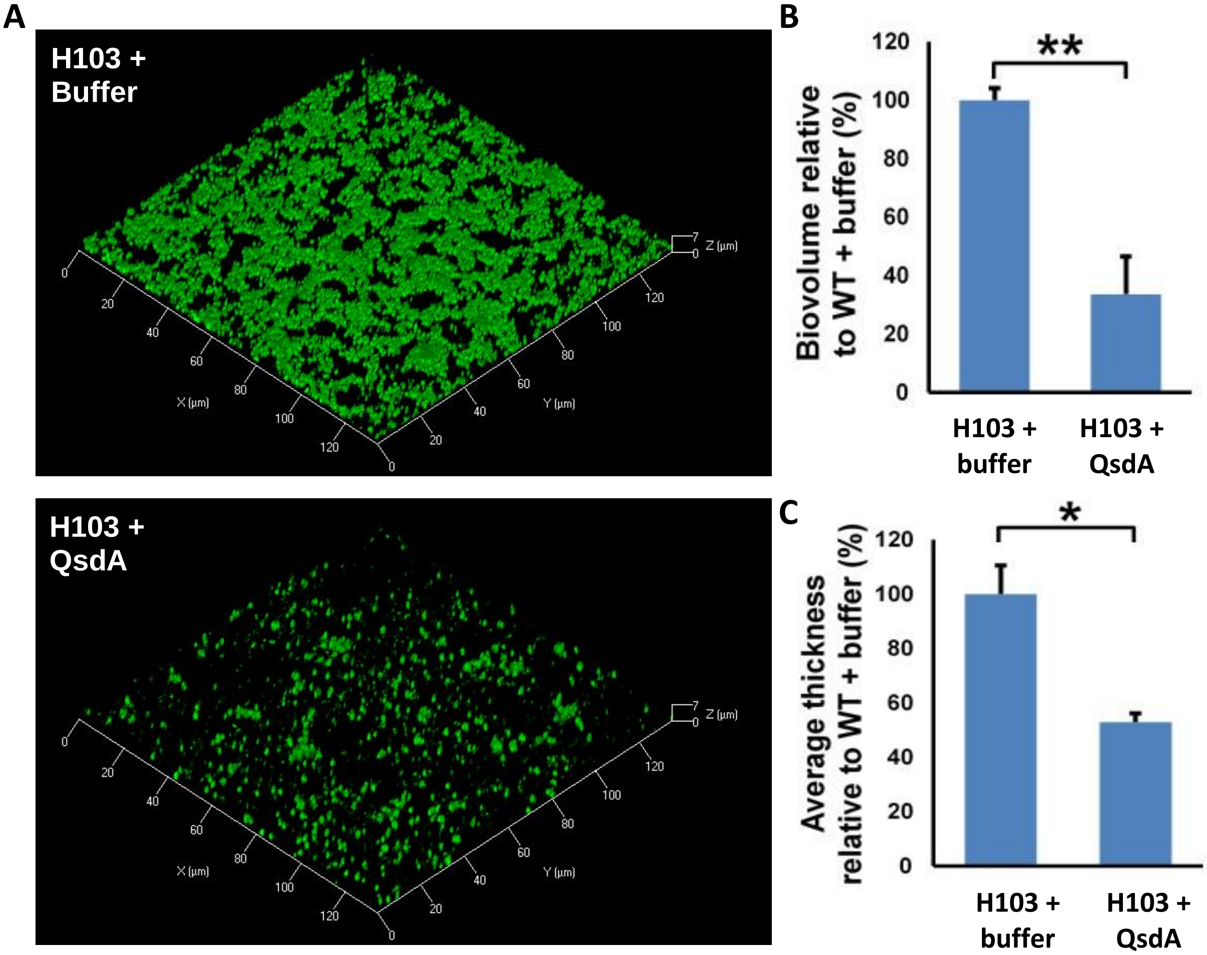

Supplementary Figure 9. Schematic representation of the experimental setup used to expose biofilm culture to volatile compounds.
(A) Exposition to Bacterial VCs. To expose biofilm to MFE01 or mutants' volatile compounds, two wells of a 24-well glass bottom plate were filled with 2 mL of LB agar (LBg). Then, 10 µL of a bacterial suspension equilibrated at OD_{580} = 10 were deposited on LBg and plates were incubated overnight prior to start biofilm culture. Each biofilm culture was adjacent to two LBg-filled wells.
(B) Exposition to pure 1-undecene. To expose biofilm to pure 1-undecene, the same protocol was used with modifications. A mixed cellulose membrane (Merk) was deposited on each LBg-filled well. A 220 µM 1-undecene solution in 100% ethanol was prepared from pure 1-undecene. 2 µL of this solution were deposited on membranes at the beginning of biofilm cultures. After 24h of incubation, 1-undecene was renewed.

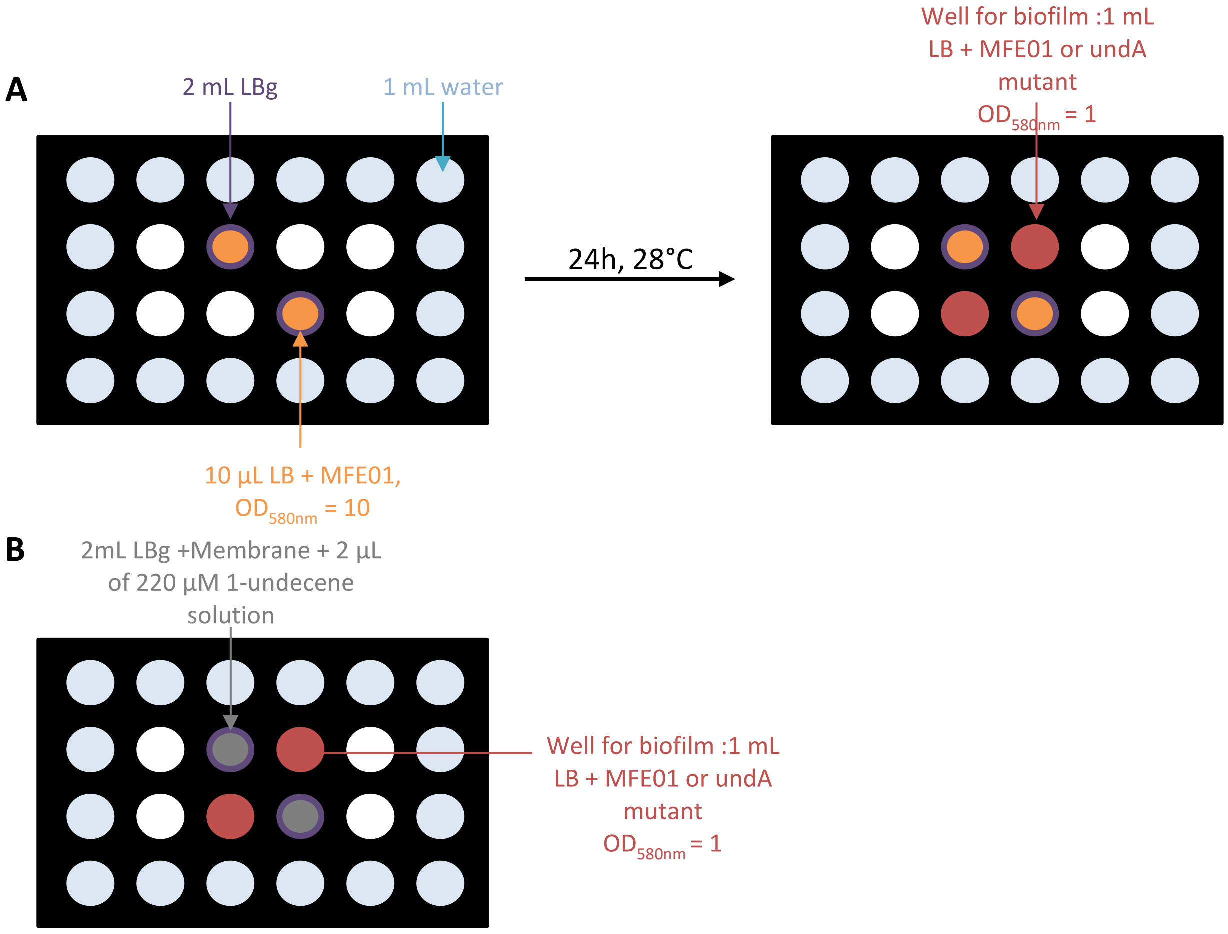

Supplement: Supplementary file 1 [file Data_Sheet_1.pdf]
